# Rapid Depletion of DIS3, EXOSC10, or XRN2 Reveals the Immediate Impact of Exoribonucleolysis on Nuclear RNA Metabolism and Transcriptional Control

**DOI:** 10.1016/j.celrep.2019.02.012

**Published:** 2019-03-05

**Authors:** Lee Davidson, Laura Francis, Ross A. Cordiner, Joshua D. Eaton, Chris Estell, Sara Macias, Javier F. Cáceres, Steven West

**Affiliations:** 1 The Living Systems Institute, University of Exeter, Stocker Rd, Exeter EX4 4QD, UK; 2 MRC Human Genetics Unit, Institute of Genetics and Molecular Medicine, Western General Hospital, University of Edinburgh, Edinburgh EH4 2XU, UK

**Keywords:** exosome, EXOSC10/Rrp6, DIS3, transcription, XRN2, non-coding RNA, degradation

## Abstract

Cell-based studies of human ribonucleases traditionally rely on methods that deplete proteins slowly. We engineered cells in which the 3′→5′ exoribonucleases of the exosome complex, DIS3 and EXOSC10, can be rapidly eliminated to assess their immediate roles in nuclear RNA biology. The loss of DIS3 has the greatest impact, causing the substantial accumulation of thousands of transcripts within 60 min. These transcripts include enhancer RNAs, promoter upstream transcripts (PROMPTs), and products of premature cleavage and polyadenylation (PCPA). These transcripts are unaffected by the rapid loss of EXOSC10, suggesting that they are rarely targeted to it. More direct detection of EXOSC10-bound transcripts revealed its substrates to prominently include short 3′ extended ribosomal and small nucleolar RNAs. Finally, the 5′→3′ exoribonuclease, XRN2, has little activity on exosome substrates, but its elimination uncovers different mechanisms for the early termination of transcription from protein-coding gene promoters.

## Highlights

• Engineered human cells for rapid inducible degradation of EXOSC10 and DIS3
• DIS3 degrades the majority of nuclear exosome substrates
• Direct targets of EXOSC10 include ribosomal and small nucleolar RNAs
• XRN2 has little activity on exosome substrates

## Graphical Abstract

**Figure:**
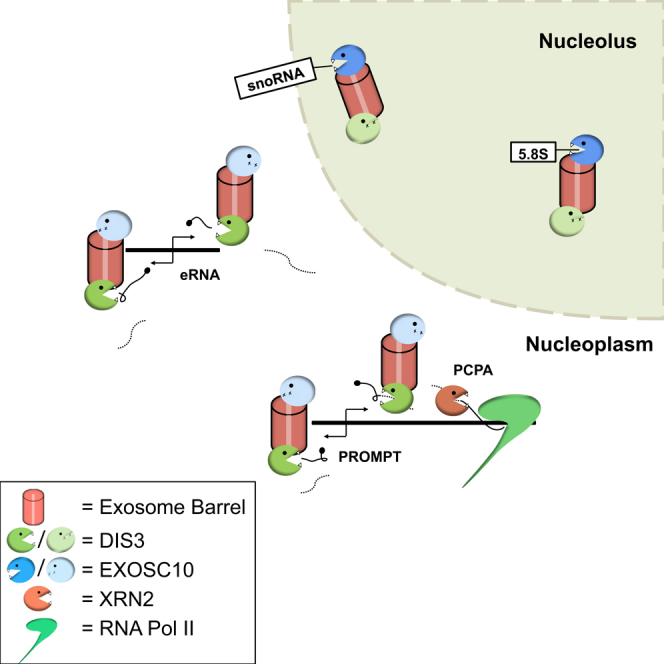

## Teaser

Using a system allowing very rapid protein depletion, Davidson et al. characterize the acute targets of the nuclear RNA degradation machinery in human cells. The thousands of substrates uncovered reveal wide ranging and immediate roles for exoribonucleases in non-coding RNA degradation, RNA processing, and early transcriptional termination.

## Introduction

The RNA exosome is a multi-subunit, 3′→5′ exoribonuclease-containing complex originally discovered as being important for rRNA processing (Mitchell et al., 1997). It also plays a crucial role in the turnover of multiple coding and non-coding (nc) transcript classes (Kilchert et al., 2016, Schmid and Jensen, 2018). Many of these transcripts, such as cryptic unstable transcripts (CUTs) in yeast or promoter upstream transcripts/upstream antisense RNAs (PROMPTs/uaRNAs) in humans, are products of antisense transcription (Flynn et al., 2011, Preker et al., 2008, Wyers et al., 2005). An additional class of ncRNAs in humans, termed enhancer RNAs (eRNAs), are produced from divergent transcription at intergenic enhancer sequence elements. Like many other pervasive transcripts, eRNAs are highly sensitive to exosome degradation (Andersson et al., 2014). More recently, products of premature cleavage and polyadenylation (PCPA) were also revealed as exosome substrates in mouse embryonic stem cells (mESCs) (Chiu et al., 2018).

The structure of the exosome is similar in yeast and humans and is composed of 9–11 key protein subunits (Gerlach et al., 2018, Januszyk and Lima, 2014, Makino et al., 2013, Weick et al., 2018). It possesses a catalytically inactive barrel structure of 9-core subunits (EXO-9), arranged as a hexamer (the PH-like ring) capped with a trimeric S1/KH ring. EXO-9 interacts with two 3′→5′ exoribonucleases: EXOSC10 (Rrp6 in budding yeast) and DIS3 (also known as Rrp44) (Makino et al., 2013). In budding yeast, DIS3 is present in both nuclear and cytoplasmic exosome complexes, but Rrp6 is found only in the nuclear complex (Allmang et al., 1999b). The composition of the exosome is more complicated in humans due to the presence of DIS3 subtypes; however, the canonical DIS3 is predominantly found within the nucleoplasm (Tomecki et al., 2010). Similar to Rrp6, EXOSC10 is nuclear and is enriched within the nucleolus (Tomecki et al., 2010). While DIS3 and the core exosome components are essential in budding yeast, cells lacking Rrp6 are viable (Allmang et al., 1999b, Briggs et al., 1998).

EXOSC10 is a member of the RNase D family and contains a DEDD-Y active site providing distributive exoribonuclease activity (Januszyk et al., 2011). DIS3 is a processive ribonuclease related to the RNase II/R family, possessing an RNB and N-terminal PIN domain, and is capable of both exoribonuclease and endoribonuclease activity (Lebreton et al., 2008, Schneider et al., 2009). When interacting with the exosome complex, Rrp6 is localized on top of the S1/KH cap, close to the entry pore leading into the central channel passing through EXO-9, whereas DIS3 is associated with the channel exit pore at the opposing pole of EXO-9 (Makino et al., 2013, Wasmuth et al., 2014). Rrp6 can widen the entry pore leading into the central channel of EXO-9 facilitating threading of RNAs through EXO-9 toward DIS3 (Wasmuth et al., 2014). RNA substrates entering the S1/KH cap can also be directed to the active site of Rrp6 for trimming and degradation. Exosome activity is further enhanced by a range of co-factors, including the helicase MTR4 (Lubas et al., 2011, Weick et al., 2018).

Genome-wide characterization of human exosome substrates have reported DIS3 as the main ribonuclease subunit responsible for degrading PROMPTs, prematurely terminated protein-coding transcripts, and eRNAs (Szczepińska et al., 2015). The targets for EXOSC10 in human cells are less well characterized, but include rRNA precursors (Macias et al., 2015, Sloan et al., 2013). In budding yeast, the active site of Rrp6 can aid in the processing of RNA substrates with more complex secondary structures, which is important during the maturation of precursor rRNAs (Fromm et al., 2017). Uncovering previously unknown RNAs has also increased our understanding of transcriptional regulation. For example, the discovery of PROMPTs helped to identify bi-directional transcription from most human promoters (Preker et al., 2008). While our study was in progress, products of PCPA were found to be stabilized by exosome loss, indicating that a proportion of truncated protein-coding RNA precursors are degraded (Chiu et al., 2018). This process is influenced by the recruitment of U1 small nuclear RNA (snRNA) to pre-mRNA and may constitute a transcriptional checkpoint. Both PROMPTs and PCPA products frequently have poly(A) signals (PASs) at their 3′ ends and possess poly(A) tails when the exosome is depleted (Almada et al., 2013, Ntini et al., 2013). As such, a PAS-dependent mechanism is proposed for attenuating their transcription.

Studies of the exosome complex in human cells usually involve protein depletion by RNAi, which is slow. The advantages of rapid, versus slower, depletion include reduced opportunities for compensatory effects and an ability to identify the most acute substrates rather than more gradual accumulation of RNA during long time periods, which could be indirect. This is also useful when inferring how frequently a process takes place, which is more difficult when protein depletion occurs during a period of days. We engineered human cells for rapid, inducible degradation of EXOSC10 or DIS3. Both catalytic components are essential, but DIS3 degrades the majority of nuclear exosome substrates. Direct detection of EXOSC10 substrates revealed a role in the maturation of small nucleolar RNAs (snoRNAs),reminiscent of the situation in budding yeast (Allmang et al., 1999a). Finally, the 5′→3′ exonuclease XRN2 showed little activity on any exosome substrate. However, it promotes the early termination of a subclass of transcription events from protein-coding genes, suggesting a variety of such mechanisms.

## Results

### Depletion of EXOSC10 or DIS3 Using the Auxin-Inducible Degron System

**Figure 1:**
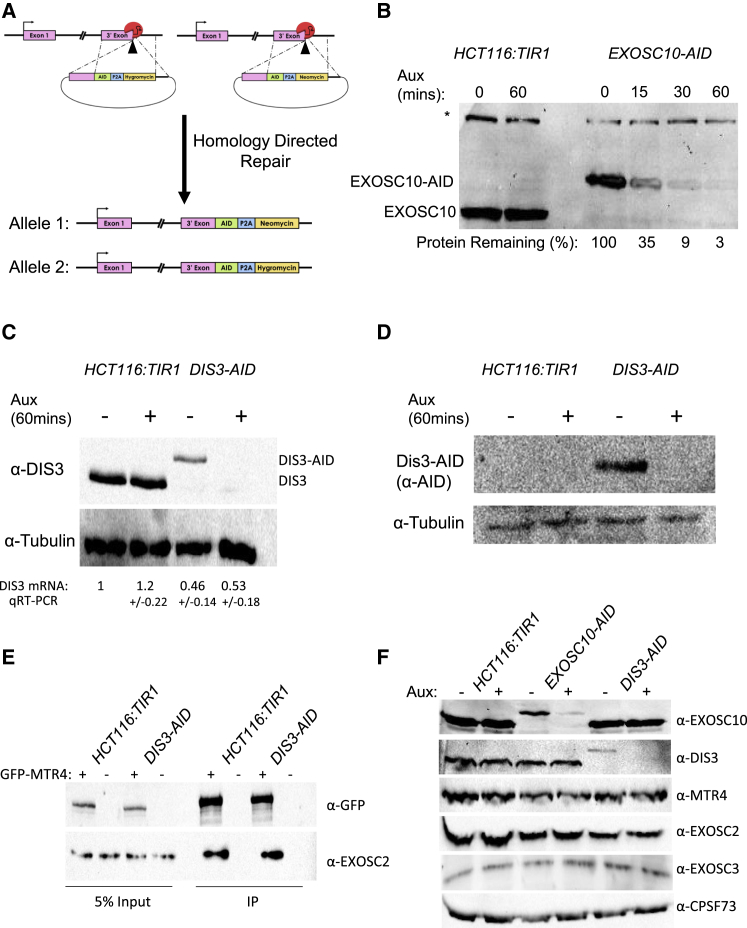
Rapid Depletion of EXOSC10 or DIS3 via the Auxin-Inducible Degron (A) Schematic showing the CRISPR strategy for modifying gene loci. Two repair cassettes were generated containing the AID tag, a P2A cleavage site, and either the hygromycin or neomycin resistance marker, followed by an SV40 PAS. These were flanked by 5′ and 3′ homology arms for the gene of interest. (B) Western blotting of EXOSC10 in either parental Tir1-expressing HCT116 (*HCT116:TIR1*) or *EXOSC10-AID* cells. A time course of auxin addition was applied to the *EXOSC10-AID* cells. Equal loading is shown by the presence of a non-specific product (^∗^) on the same blot. (C) Western blotting of DIS3 in either *HCT116:TIR1* or *DIS3-AID* cells treated or not treated for 60 min with auxin. Tubulin was probed for as a loading control. Quantitative reverse transcription and PCR -derived levels of DIS3 mRNA also shown (including standard deviations [SDs]), obtained following normalization to glyceraldehyde 3-phosphate dehydrogenase (GAPDH) levels. (D) Western blotting of DIS3 in either *HCT116:TIR1* or *DIS3-AID* cells treated or not treated for 60 min with auxin using an antibody to the AID tag. Tubulin was probed for as a loading control. (E) Co-immunoprecipitation (coIP) of GFP-MTR4 and EXOSC2 in *HCT116:TIR1* or *DIS3-AID* cells. Input (5%) and IP are shown. Blots were probed with α-GFP (to detect GFP-MTR4) or α-EXOSC2. (F) Western blotting of EXOSC10, DIS3, MTR4, EXOSC2, EXOSC3, and as a loading control, CPSF73 in *HCT116:TRI1*, *DIS3-AID*, or *EXOSC10-AID* cells treated or not treated with auxin (1 h). Due to the similar size of some of these proteins, multiple blots were probed rather than using stripping. Equal loading was confirmed by loading control or ponceau. Pictures of individual blots are deposited at Mendeley (see Method Details).

The auxin-inducible degron (AID) system allows the rapid elimination of AID-tagged proteins upon the addition of auxin to cell culture media (Nishimura et al., 2009). CRISPR/Cas9 was used to C-terminally tag *EXOSC10* or *DIS3* with an AID (Figure 1A). Hygromycin or neomycin resistance markers were incorporated into the cassettes for homology directed repair (HDR) so that bi-allelic modification could be selected for (Eaton et al., 2018). A P2A site, between the AID and drug markers, ensured their separation via peptide cleavage during translation (Kim et al., 2011). This system requires expression of the plant E3 ubiquitin ligase, Tir1, which we previously introduced stably into HCT116 cells, chosen for their diploid karyotype.

Western blotting confirmed successful AID tagging of *EXOSC10* as a species of the predicted molecular weight of EXOSC10-AID was detected in *EXOSC10-AID* cells with native-sized protein absent (Figure 1B). This was confirmed by the exclusive detection of native-sized EXOSC10 in parental *HCT116:TIR1* cells. A time course of auxin addition demonstrated rapid depletion of EXOSC10-AID, which was reduced by ∼97% after 60 min with native EXOSC10 insensitive to auxin. Western blotting also showed the exclusive presence of DIS3-AID in *DIS3-AID* cells and its depletion upon auxin treatment (Figure 1C). DIS3-AID is expressed at lower levels than native DIS3, and quantitative reverse transcription and PCR showed that there is a ∼50% reduction in spliced DIS3-AID mRNA (Figure 1C). A monoclonal antibody to the AID tag also detected DIS3-AID, which is absent from *HCT116:TIR1* cells and eliminated within 60 min of auxin treatment (Figure 1D). Although DIS3-AID is expressed at lower levels than native DIS3, it does not limit the association of essential co-factors with the exosome core, as we observed equal co-immunoprecipitation of EXOSC2 with GFP-MTR4 in *DIS3-AID* and parental cells (Figure 1E).

To demonstrate the specificity of EXOSC10-AID and DIS3-AID depletion, we monitored the levels of several exosome components (EXOSC10, DIS3, EXOSC2, EXOSC3, and MTR4) in parental, *DIS3-AID*, and *EXOSC10-AID* cells treated or not treated with auxin (Figure 1F). Tagging *EXOSC10* or *DIS3* had no impact on the levels of other exosome factors in the absence of auxin. Auxin treatment specifically eliminated the tagged factors without co-depleting other proteins.

### Rapid Depletion of EXOSC10-AID or DIS3-AID Leads to Accumulation of Unstable RNAs

**Figure 2:**
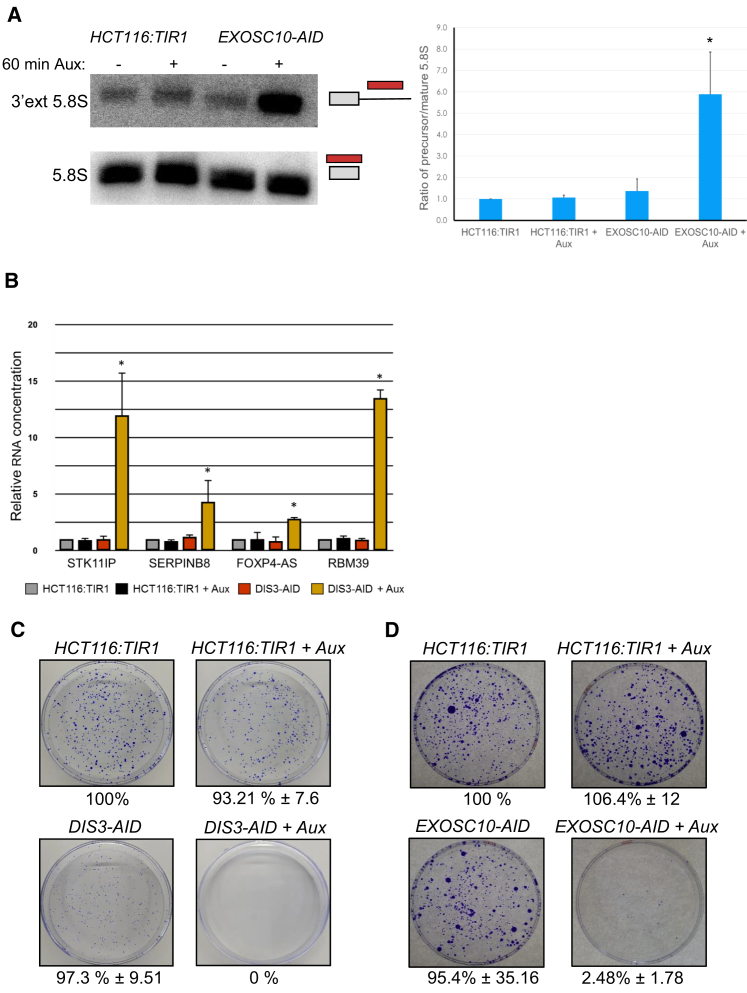
Effects of DIS3/EXOSC10 Depletion on RNA Substrates and Cell Viability (A) Northern blot analysis of mature (bottom) and 3′ extended (top) 5.8S rRNA performed in *HCT116:TIR1* cells and *EXOSC10-AID* cells treated or not treated with auxin. Bar graph shows quantitation expressed as a ratio of extended to mature species. n = 3. ^∗^p < 0.05. Error bars are SDs. (B) Quantitative reverse transcription and PCR detection of STK11IP, SERPINB8, FOXP4-AS, and RBM39 PROMPTs in *HCT116:TIR1* cells and *DIS3-AID* cells treated or not treated with auxin (1 h). Quantitation is expressed as relative RNA level compared to that found in non-auxin-treated *HCT116:TIR1* cells after normalizing to ACTB RNA. n = 3. ^∗^p < 0.05. Error bars are SDs. (C) Colony formation assay for HCT116:TIR1 cells and *DIS3-AID* cells grown with or without auxin. Number of colonies expressed as a percentage of those forming from *HCT116:TIR1* cells grown in the absence of auxin. Values show averages and SDs from n = 3. (D) As in (C), but for *HCT116:TIR1* and *EXOSC10-AID* cells.

We next tested the effects of eliminating EXOSC10-AID or DIS3-AID on some of their known substrates. To check for any adverse effects of auxin addition or the AID tag, we added the parental *HCT116:TIR1* cells to the experimental series. Depletion of EXOSC10 has been shown to stabilize a short 3′ extended version of the 5.8S rRNA (Allmang et al., 1999b, Briggs et al., 1998, Schilders et al., 2007). We performed northern blotting on total RNA isolated from *EXOSC10-AID* cells treated or not treated with auxin for 60 min and probed blots for either mature or 3′ extended 5.8S rRNA (Figure 2A). 3′ extended 5.8S rRNA was weakly detected in treated and untreated *HCT116:TIR1* cells and in untreated *EXOSC10-AID* cells. However, auxin treatment of *EXOSC10-AID* cells induced a strong increase in its levels. As such, acute depletion of EXOSC10 is sufficient to reveal its RNA substrates with no apparent adverse effect of the AID tag.

For DIS3, we analyzed the levels of 3 PROMPTs (STK11IP, SERPINB8, and RBM39) and 1 antisense transcript (FOXP4-AS). This was done in *DIS3-AID* cells treated or not treated with auxin (60 min) and in *HCT116:TIR1* cells grown under the same conditions (Figure 2B). Quantitative reverse transcription and PCR showed no auxin-dependent changes in *HCT116:TIR1* cells, as expected. PROMPT levels were similarly low in *DIS3-AID* cells untreated with auxin, demonstrating that DIS3-AID is sufficient for their normal turnover. However, auxin treatment of *DIS3-AID* cells results in a large increase in all cases, confirming the effectiveness of this system.

### DIS3 and EXOSC10 Are Essential in Human Cells

We next tested whether EXOSC10 and DIS3 are required for cell viability. Colony formation assays were performed on *EXOSC10-AID* or *DIS3-AID* cells grown in the presence and absence of auxin and on *HCT116:TIR1* cells under the same conditions. *HCT116:TIR1* cells formed a similar number of colonies in the presence and absence of auxin, demonstrating no adverse effects of auxin on viability (Figure 2C). *DIS3-AID* cells formed as many colonies as *HCT116:TIR1* cells when auxin was omitted, but their smaller size highlights a slight reduction in growth. No *DIS3-AID* cell colonies formed in the presence of auxin, showing that DIS3 is essential. *EXOSC10-AID* cells showed no statistically significant defect in colony formation in the absence of auxin, compared to *HCT116:TIR1* cells (Figure 2D). However, auxin prevented the formation of *EXOSC10-AID* cell colonies, showing that EXOSC10 is essential. This contrasts with budding yeast, in which Δ*rrp6* cells are viable (Allmang et al., 1999b).

### Nuclear RNA-Seq Analysis following EXOSC10-AID or DIS3-AID Elimination

**Figure 3:**
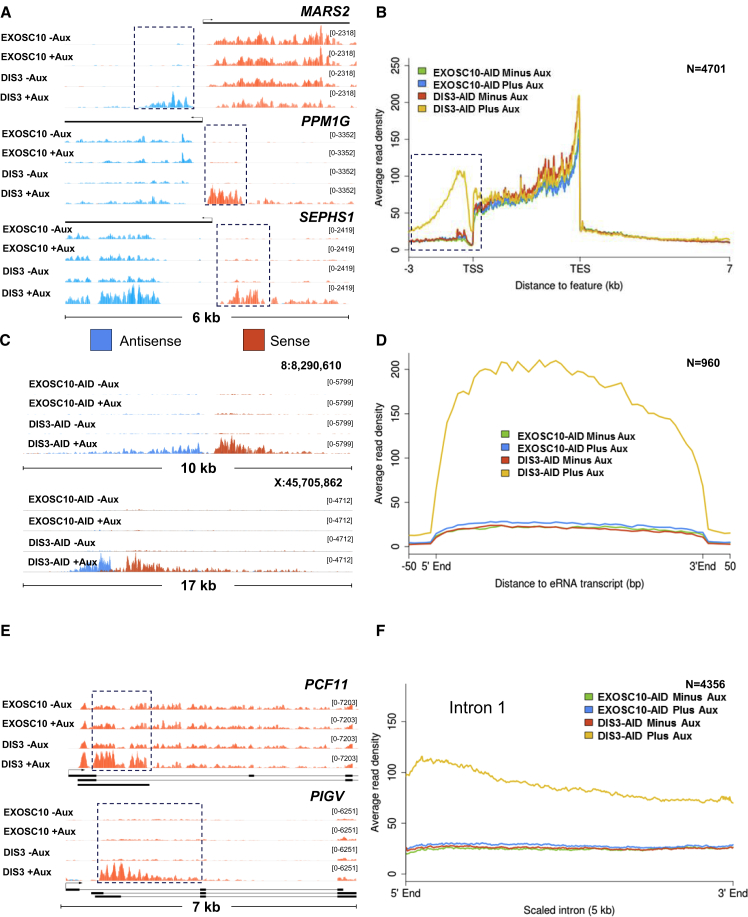
Global Analysis of the Effects of EXOSC10 or DIS3 Loss (A) Integrative genome viewer (IGV) browser tracks of MARS2, PPM1G, and SEPHS1 PROMPT transcripts (boxed) in *EXOSC10-AID* and *DIS3-AID* cells treated or not treated with auxin. y axis units are reads per kilobase per million (RPKM) mapped. (B) Metagene plot of coding and non-coding genes in *EXOSC10-AID* and *DIS3-AID* cells treated or not treated with auxin. DIS3 loss shows a strong effect on PROMPT regions (boxed). (C) IGV browser tracks of 2 eRNA regions in *EXOSC10-AID* and *DIS3-AID* cells treated or not treated with auxin. y axis units are RPKM. (D) Metagene plot of all eRNA-expressing regions in *EXOSC10-AID* and *DIS3-AID* cells treated or not treated with auxin. (E) IGV browser tracks of *PCF11* and *PIGV* in *EXOSC10-AID* and *DIS3-AID* cells treated or not treated with auxin. Both show strong upregulation of 5′ pre-mRNA upon loss of DIS3 (boxed). y axis units are RPKM. (F) Metagene plot of all first introns in *EXOSC10-AID* and *DIS3-AID* cells treated or not treated with auxin.

We next analyzed the immediate impact of EXOSC10 and DIS3 loss more globally. Nuclear RNA was extracted from *EXOSC10-AID* or *DIS3-AID* cells that had been treated or not treated with auxin for 1 h and performed RNA sequencing (RNA-seq). Nuclear RNA was chosen, as we anticipated most exosome substrates to be enriched in the nucleus. We first analyzed PROMPTs and found an obvious accumulation upon the loss of DIS3 (Figure 3A). Metagene analysis shows that PROMPTs accumulate at thousands of genes when DIS3 is absent (Figure 3B). The global increase in PROMPT levels within just 60 min of auxin treatment underscores their acute instability. Further examination of the metaplot in Figure 3B revealed no impact of either exosome subunit on the stability of 3′ flanking region RNAs, consistent with our finding that these species are XRN2 substrates (Eaton et al., 2018). Acute depletion of EXOSC10 had no effect on PROMPT transcripts, suggesting that they are not its immediate substrates.

Hundreds of intergenic transcripts were also seen upon DIS3 elimination, which were barely detectable in the absence of auxin. We presume that these are eRNAs because separating sequencing reads into sense and antisense strands showed their bidirectionality (Figure 3C). Moreover, these regions have high H3K4me1 versus H3K4me3 modified chromatin at their promoter regions, as do enhancers (Andersson et al., 2014, Core et al., 2014, Heintzman et al., 2007) (Figures S1A and S1B). A metagene analysis of these transcripts confirmed the generality of the DIS3 effect and, as with PROMPTs, shows that they are generally not substrates for EXOSC10 (Figure 3D). Our experiment again highlights the acute instability of eRNAs and straightforward uncovering of almost 1,000 examples upon DIS3 loss. This is a similar number to what has been reported in other mammalian cells when the exosome was depleted during several days (Pefanis et al., 2015).

Protein-coding promoters also produce a variety of exosome substrates in the sense direction, some of which are generated by PCPA (Chiu et al., 2018, Iasillo et al., 2017, Ogami et al., 2017). Truncated pre-mRNA products are readily apparent in our data following rapid depletion of DIS3, but not when EXOSC10 is lost (Figure 3E). A prominent example is observed for PCF11 pre-mRNA, which is subject to PCPA in mESCs (Chiu et al., 2018). To test the generality of DIS3-mediated turnover of truncated pre-mRNAs, we generated a metagene plot covering the first intron of genes (Figure 3F). This showed an obvious enhancement of intron 1 levels in cells depleted of DIS3, with no effect of EXOSC10 loss observed. This effect is still evident when intron read counts are normalized to those over the first exon, but is diminished over the second or fourth intron (Figures S1C–S1E). The robust accumulation of such RNAs within minutes of DIS3 loss is an important observation that underscores the high frequency of attenuated transcription. All of the above DIS3 effects were confirmed in an independent biological RNA-seq replicate (Figure S2).

### There Is Little Redundancy between EXOSC10 and DIS3 Activity on Nucleoplasmic PROMPTs

**Figure 4:**
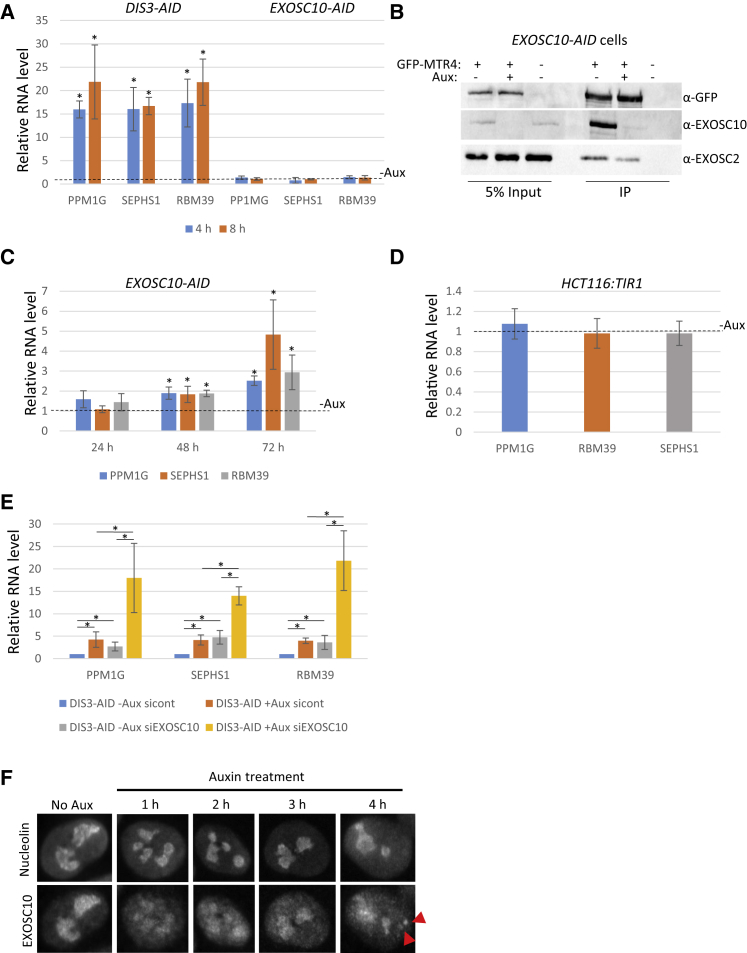
Analysis of Redundancy between EXOSC10 and DIS3 (A) Quantitative reverse transcription and PCR analysis of PPM1G, SEPHS1, and RBM39 PROMPTs in *DIS3-AID* or *EXOSC10-AID* cells treated or not treated with auxin for 4 and 8 h. Levels are expressed as fold change compared to untreated cells (dotted line) following normalization to GAPDH mRNA. n = 3. ^∗^p < 0.05 for differences concluded on in the text. Error bars show SDs. (B) coIP of EXOSC10 or EXOSC2 with GFP-MTR4 in *EXOSC10-AID* cells treated or not treated with auxin (2 h). Input and IP are shown with blots probed with α-GFP (to detect GFP-MTR4), α-EXOSC10, or α-EXOSC2. (C) Quantitative reverse transcription and PCR analysis of PPM1G, SEPHS1, and RBM39 PROMPTs in *EXOSC10-AID* cells treated or not treated with auxin for 24, 48, or 72 h. Levels are expressed as fold change compared to untreated cells (indicated by dotted line) following normalization to GAPDH mRNA. n = 3. ^∗^p < 0.05. Error bars show SDs. (D) Quantitative reverse transcription and PCR analysis of PPM1G, SEPHS1, and RBM39 PROMPTs in *HCT116:TIR1* cells treated or not treated with auxin for 72 h. Levels are expressed as fold change compared to untreated cells (indicated by dotted line) following normalization to GAPDH mRNA. n = 3. Error bars show SDs. (E) Quantitative reverse transcription and PCR analysis of PPM1G, SEPHS1, and RBM39 PROMPTs in *DIS3-AID* cells transfected with control or EXOSC10-specific siRNAs before treatment or no treatment with auxin (1 h). Levels are expressed as fold change compared to control siRNA transfected cells not treated with auxin following normalization to GAPDH mRNA. n = 4. ^∗^p < 0.05 for differences concluded on in the text. Error bars show SDs. (F) EXOSC10 immunofluorescence in untreated *DIS3-AID* cells or the same cells treated with auxin for 1, 2, 3, or 4 h. The same cells stained with nucleolin are also shown. The red arrowheads show EXOSC10 puncta that do not overlap with nucleolin signal.

A striking outcome of our RNA-seq data is the lack of effect of EXOSC10 on the thousands of nucleoplasmic exosome substrates degraded by DIS3. In contrast, depletion of EXOSC10 by RNAi often affects nucleoplasmic transcripts, and co-depletion of EXOSC10 and DIS3 can produce synergistic effects that imply some redundancy (Lubas et al., 2011, Tomecki et al., 2010). To analyze the effects of EXOSC10 on nucleoplasmic substrates more closely, we performed a more extended time course of auxin treatment (4 and 8 h) in *EXOSC10-AID* or *DIS3-AID* cells, followed by the quantitation of SEPHS1, RBM39, and PPM1G PROMPTs (Figure 4A). While DIS3-AID loss increases the levels of all 3 transcripts, none were significantly affected by the absence of EXOSC10-AID. MTR4 associates with the exosome core whether EXOSC10-AID is present or not, supporting the existence of functional complexes, even when EXOSC10 is absent (Figure 4B). We next treated *EXOSC10-AID* cells for 24, 48, or 72 h with auxin, which revealed a mild increase in PROMPTs at longer time points (Figure 4C). As EXOSC10 effects require long-term protein depletion, this increase could be due to the indirect consequences of its loss or reflective of very occasional roles in PROMPT turnover. This is not an indirect effect of auxin, as PROMPT levels were unaffected in parental cells after 72 h of treatment (Figure 4D).

The absence of acute effects of EXOSC10 on PROMPTs argues that DIS3 degrades them in its absence. To test this, *DIS3-AID* cells were transfected with control or EXOSC10-specific small interfering RNAs (siRNAs) before treatment or no treatment with auxin. Quantitative reverse transcription and PCR was then used to analyze the levels of SEPHS1, RBM39, and PPM1G PROMPTs (Figure 4E). DIS3 elimination from control siRNA-treated cells caused the upregulation of each PROMPT as expected. For RBM39, this effect was generally not as large as in Figure 2B, which may result from the additional perturbation caused by RNAi. EXOSC10 depletion caused an increase in PROMPT levels, even in the presence of DIS3-AID, which is consistent with the small effect of EXOSC10-AID loss at long time points of auxin treatment. Auxin treatment of EXOSC10-depleted *DIS3-AID* cells revealed a larger enhancement of PROMPT levels than the depletion of either protein alone. As such, although EXOSC10 plays little role in PROMPT RNA degradation under normal circumstances, its presence may be more important when DIS3 levels are very low.

### DIS3 Loss Disrupts Focused Nucleolar Localization of EXOSC10

To understand why low DIS3 levels may lead to degradation of some nucleoplasmic exosome substrates by EXOSC10, we monitored its localization in *DIS3-AID* cells treated or not treated with auxin over a time course (Figure 4F). As previously reported (Lubas et al., 2011, Tomecki et al., 2010), EXOSC10 is nucleolar enriched as shown by co-localization with nucleolin. DIS3-AID loss resulted in less focused nucleolar localization of EXOSC10 (also see Figure S3A). This was not due to a breakdown of nucleoli, as nucleolin signal showed little alteration in the same cells. Furthermore, at extended time points of DIS3-AID loss, we observed nucleoplasmic puncta of EXOSC10 in ∼25% of cells that do not overlap with nucleolin signal. EXOSC10 localization in *DIS3-AID* cells is identical to the parental cell line, and analysis of wider fields of cells confirmed the generality of the effects (Figures S3B and S3C). We conclude that DIS3-AID loss disrupts the normally focused nucleolar localization of EXOSC10, which may allow it to engage with nucleoplasmic substrates and potentially explain the synergistic effect of EXOSC10 and DIS3 co-depletion on PROMPTs.

### EXOSC10 Is Involved in 3′ Trimming of Pre-rRNA and Pre-snoRNA Transcripts

We next wanted to identify specific substrates of EXOSC10 and used individual-nucleotide resolution UV crosslinking and immunoprecipitation (iCLIP) to detect transcripts to which it directly binds. We complemented previous iCLIP data, generated using functional EXOSC10 (EXOSC10^WT^) in HEK293T cells (Macias et al., 2015), with iCLIP using a catalytically dead version of EXOSC10 (EXOSC10^CAT^) also expressed in HEK293T cells. EXOSC10^CAT^ contains a single substitution (D313N) previously shown to abolish EXOSC10 activity (Januszyk et al., 2011). We reasoned that EXOSC10^CAT^ would associate more stably with EXOSC10 substrates and facilitate their detection.

**Figure 5:**
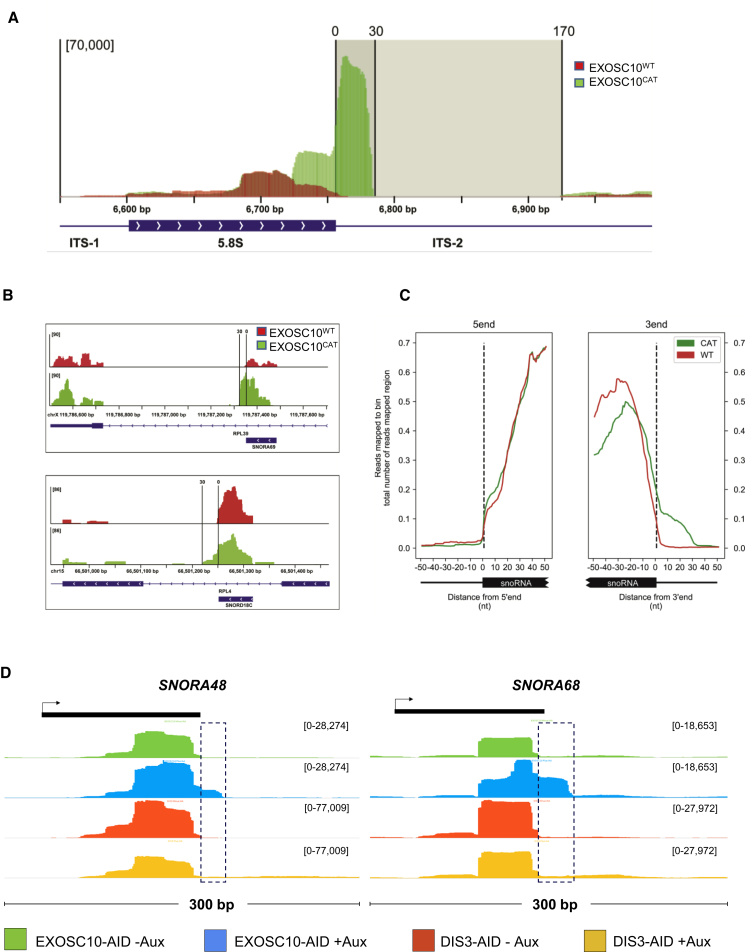
Direct Detection of EXOSC10 Substrates by iCLIP (A) iCLIP trace of 5.8S rRNA locus obtained from EXOSC10^WT^ and EXOSC10^CAT^ samples. There is a clear enrichment of reads for the EXOSC10^CAT^ sample showing a 30-nt “footprint” immediately beyond the 5.8S gene (indicated by vertical lines). y axis units are reads per million mapped. (B) iCLIP traces of *SNORA69* and *SNORD18C* genes obtained from EXOSC10^WT^ and EXOSC10^CAT^ samples. There is strong enrichment of reads for the EXOSC10^CAT^ sample showing a 30 nucleotide footprint immediately beyond each gene. y axis units are reads per million mapped. (C) Metagene plots of iCLIP reads over the 5′ or 3′ regions of snoRNA genes in EXOSC10^WT^ and EXOSC10^CAT^ samples. There is a clear 30-nt footprint immediately 3′ of snoRNA genes. (D) IGV browser tracks of *SNORA48* and *SNORA68* genes in *EXOSC10-AID* and *DIS3-AID* cells treated or not treated with auxin. These show upregulation of short 3′ extended versions of each (boxed) in auxin-treated *EXOSC10-AID* cells. y axis units are RPKM.

As EXOSC10 loss leads to the accumulation of 3′ extended 5.8S rRNA (Figure 2A), we validated our iCLIP data by first assessing this potential substrate. There was a strong iCLIP signal specifically at this site in EXOSC10^CAT^ samples, which had 33-fold more reads than EXOSC10^WT^ mapping within a 30-nt window downstream of 5.8S (Figure 5A). This large read density seen in EXOSC10^CAT^ indicates that the catalytic mutant blocks the processing of pre-5.8S and underscores it as a *bona fide* EXOSC10 substrate. The expression of inactive EXOSC10 in *EXOSC10-AID* cells consistently enhances the levels of extended 5.8S RNA in a dominant-negative fashion (Figures S4A and S4B). Read density rapidly drops beyond 30 nt downstream of the annotated end of 5.8S rRNA, suggesting that EXOSC10 is required only for the final nuclear trimming step. This indicates a ribonuclease switch and is consistent with reconstituted 5.8S rRNA maturation in budding yeast, during which DIS3 processing is sterically inhibited by the exosome core, necessitating handover to Rrp6 (Fromm et al., 2017, Makino et al., 2015). Analysis of the entire 45S rDNA showed significant CLIP density over the 5′ external transcribed spacer (ETS) in both EXOSC10^WT^ and EXOSC10^CAT^ (Figure S4C).

We reasoned that the 30-nt “footprint” downstream of the 5.8S rRNA, seen in EXOSC10^CAT^ samples, can identify other RNAs that are subject to final processing by EXOSC10. Obvious ∼30-nt footprints of CLIP density were identified in 3′ flanking regions of snoRNAs, with examples shown for SNORA69 and SNORD18C in Figure 5B. Metagene analyses of the average distribution of EXOSC10 iCLIP reads over annotated snoRNAs indicate that EXOSC10 engages in processing pre-snoRNAs that are extended at their 3′ ends by ∼30 nt due to the specific enrichment of CLIP density exclusively seen in the EXOSC10^CAT^ iCLIP dataset (Figure 5C). A majority of snoRNAs in both the SNORD and SNORA classes showed this signature of EXOSC10^CAT^ binding (Figure S5A). Analysis of our RNA-seq data independently revealed examples in which short extended snoRNA precursors are specifically stabilized by EXOSC10 loss (Figure 5D). Overall, these data identify short 3′ extended RNA precursors as EXOSC10 substrates. The implication of EXOSC10 in human snoRNA processing highlights conservation with budding yeast in which Rrp6 performs a similar 3′ trimming step (Allmang et al., 1999a). We also noted examples in which longer 3′ snoRNA extensions were seen in the absence of DIS3, which is consistent with a ribonuclease handover and previous photoactivatable ribonucleoside (PAR)-CLIP analysis (Szczepińska et al., 2015) (Figure S5B). Finally, unlike for 3′ extended snoRNA and 5.8S rRNA, PROMPT and eRNA reads were not enriched in the EXOSC10^CAT^ experiment, and the exclusive expression of inactive EXOSC10 did not stabilize PROMPTs (Figures S5C and S5D). This further demonstrates that they are not usually EXOSC10 substrates.

### Analysis of XRN2 Regulation of Exosome-Targeted Transcripts

Transcripts can also be degraded from their 5′ end, with XRN2 being the major nuclear 5′→3′ exoribonuclease and having a prominent role in transcriptional termination (Eaton et al., 2018). Although RNAi has also been used to study XRN2, it may not reveal its full repertoire of functions, as we suggested previously by engineering *XRN2-AID* cells (Eaton et al., 2018). To more accurately assess the impact of XRN2 on PROMPT and eRNA degradation, we analyzed our previously published nuclear RNA-seq from *XRN2-AID* cells in which XRN2 is eliminated within 60 min of auxin treatment (Figure S6). There was no general impact of XRN2 elimination on either of these transcript classes, indicating that they are not its substrates.

**Figure 6:**
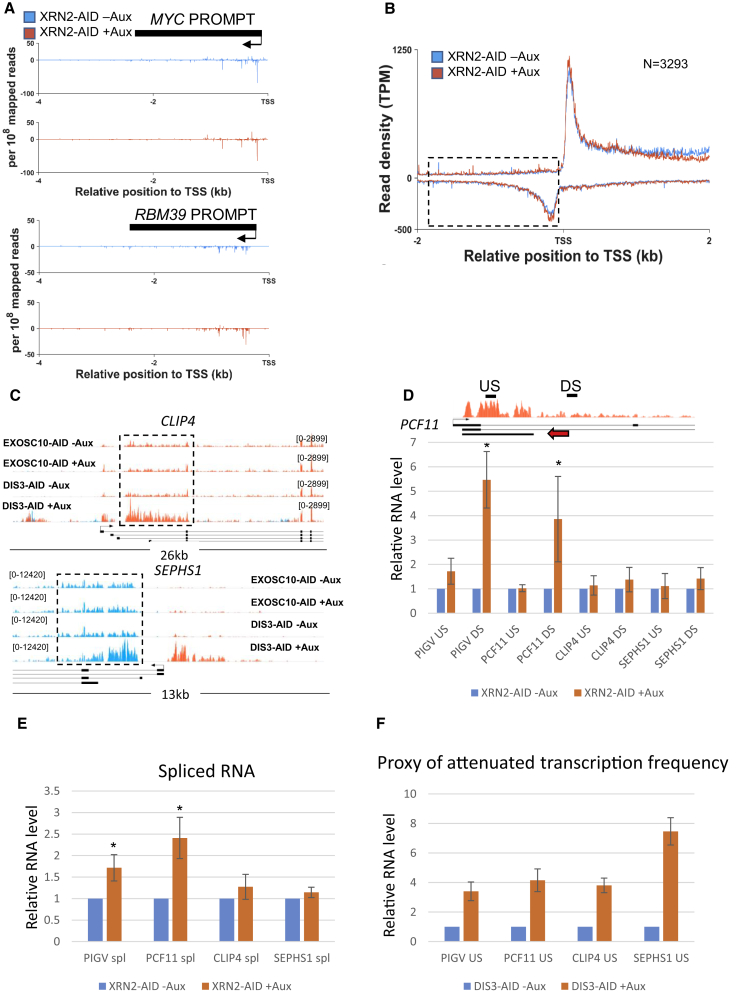
Effects of Rapid XRN2 Loss on Exosome Substrates and Early Transcriptional Termination (A) *MYC* and *RBM39* PROMPT region tracks in mNET-seq data obtained from *XRN2-AID* cells treated or not treated with auxin. y axes show signals per 10^8^ mapped reads. (B) Metagene analysis of PROMPT regions (boxed) in mNET-seq data obtained from *XRN2-AID* cells treated or not treated with auxin. TPM, transcripts per million. Signal below zero on the y axis represents antisense PROMPT transcription. (C) Gene tracks of *CLIP4* and *SEPHS1* attenuated transcription in *EXOSC10-AID* or *DIS3-AID* cells treated or not treated with auxin (1 h). Truncated RNAs stabilized by DIS3 loss are boxed. y axis shows RPKM. (D) Quantitative reverse transcription and PCR analysis of premature transcriptional termination at *PCF11, PIGV, CLIP4*, and *SEPHS1* genes in 4sU-labeled RNA from *XRN2-AID* cells treated or not treated with auxin (1 h). A gene track for *PCF11* shows DIS3-stabilized products together with approximate primer positions. Red arrow denotes annotated PCF11 PCPA product. The same primer position principles apply to the other 3 genes tested. Graph shows quantitation where values are plotted relative to those in untreated *XRN2-AID* cells following normalization to spliced GAPDH mRNA levels. n ≥ 3. ^∗^p < 0.05. Error bars show SDs. (E) Quantitative reverse transcription and PCR analysis of spliced PCF11, PIGV, CLIP4, and SEPHS1 mRNA in 4sU-labeled RNA extracted from *XRN2-AID* cells treated or not treated with auxin (1 h). Graph shows quantitation where values are plotted relative to those in untreated *XRN2-AID* cells following normalization to spliced GAPDH mRNA levels. n ≥ 3. ^∗^p < 0.05. Error bars show SDs. (F) Quantitative reverse transcription and PCR quantitation of the DIS3 effect on truncated PCF11, PIGV, CLIP4, and SEPHS1 transcripts determined in *DIS3-AID* cells treated or not treated with auxin (1 h). Graph shows quantitation where values are plotted relative to those in untreated *DIS3-AID* cells following normalization to spliced GAPDH mRNA levels. n ≥ 3. Error bars show SDs.

The termination of exosome substrates described here is poorly understood, but the *XRN2-AID* cell line allows an assessment of its role in the process. Accordingly, we analyzed PROMPT regions in mammalian native elongating transcript sequencing (mNET-seq) data that we previously generated in *XRN2-AID* cells (Eaton et al., 2018). mNET-seq analyses the position of RNA polymerase at single-nucleotide resolution by sequencing the 3′ end of RNA from within its active site (Nojima et al., 2015). A comparison of typical PROMPTs (*MYC* and *RBM39*) showed nascent transcription over these regions that terminated within ∼1.5 kb of the respective promoters (Figure 6A). XRN2 elimination caused neither more reads over the termination region nor additional reads beyond it. More general analysis of the XRN2 impact on PROMPT termination revealed only a very slight increase in signal at the 5′-most positions (also visible in the sense direction) (Figure 6B). Therefore, extended PROMPT transcription is not generally apparent in the absence of XRN2. RNA-seq consistently revealed no general effect of XRN2 loss on PROMPT levels (Figures S6A and S6B).

We also show that protein-coding genes produce exosome substrates in the sense direction (Figures 3E and 3F), and we tested the impact of XRN2 on the termination of these products. This analysis was performed on 4 truncated transcripts at the *PIGV*, *PCF11*, *CLIP4*, and *SEPHS1* genes (Figure 3E demonstrates the DIS3 effect for PCF11 and PIGV with CLIP4 and SEPHS1 data in Figure 6C). PCF11 was chosen as it is subject to PCPA in mESCs and has an annotated PCPA site in humans (Ensembl I.D.: ENST00000624931.1; Chiu et al., 2018) with the other 3 genes chosen at random. As truncated transcripts overlap with full-length transcription, we labeled nascent transcripts for 30 min with 4-thiouridine (4sU) following treatment or no treatment with auxin. 4sU-labeled RNA was then captured via biotinylation and streptavidin beads, isolating it from material that existed before the elimination of XRN2. Quantitative reverse transcription and PCR was then performed using a primer pair within the DIS3-stabilized region (upstream [US]) and another downstream (DS) of it (Figure 6D). XRN2 loss induced a significant increase in RNA downstream of the DIS3-stabilized region for PIGV and PCF11, but not for SEPHS1 or CLIP4.

Premature termination may constitute a dead-end pathway or it could compete with full-length transcription. To distinguish these possibilities, primers were designed to detect downstream splicing events in PCF11, PIGV, CLIP4, or SEPHS1 mRNAs in 4sU-labeled RNA isolated from *XRN2-AID* cells treated or not treated with auxin (Figure 6E). XRN2 depletion significantly increased the level of spliced mRNA from *PCF11* and *PIGV*, suggesting that some transcripts escaping PCPA-mediated termination are not dead-end products. However, spliced SEPHS1 or CLIP4 mRNA were unaffected by XRN2 loss, in line with its lack of impact on their attenuated transcription.

Finally, the apparent difference in sensitivity of early termination to XRN2 may be influenced by the frequency of attenuated transcription in each case. To assess this, attenuated SEPHS1, CLIP4, PIGV, and PCF11 transcripts were assayed by quantitative reverse transcription and PCR in *DIS3-AID* cells treated or not treated with auxin (Figure 6F). All 4 transcripts accumulated robustly on the loss of DIS3, demonstrating similarly frequent attenuation of transcription, with SEPHS1 showing the largest effect. As such, the insensitivity of SEPHS1 and CLIP4 early termination to XRN2 is not correlated with less frequent attenuation of transcription compared to PCF11 and PIGV. We conclude that DIS3 is involved in the widespread degradation of attenuated transcripts from protein-coding genes that fall into subtly different classes. We have distinguished some of these on the basis of their sensitivity to XRN2-dependent termination.

## Discussion

We have engineered conditional depletion of DIS3, EXOSC10, or XRN2 to assess their immediate impact on RNA metabolism. The rapid depletion achieved provides important insights that complement previous RNAi approaches. Timescales of minutes versus days have the obvious advantage that transcripts are less likely to appear through secondary effects. Moreover, an accumulation of RNA within minutes demonstrates constant turnover in a way that is more difficult to infer by RNAi, during which accumulation may be gradual. It also highlights acute substrates versus those that are only apparent after long periods of protein depletion, as exemplified by the effect of EXOSC10 on PROMPT levels.

We were initially concerned that the low levels of DIS3-AID may prove problematic for assaying the impact of its loss. However, several observations mitigate this concern. First, although DIS3 is essential, *DIS3-AID* cells produce as many colonies as *HCT116:TIR1* cells, although they are smaller. Second, *DIS3-AID* cells have the same levels of DIS3 substrates as *HCT116:TIR1* cells when auxin is not used. Third, DIS3 substrates do not accumulate upon the rapid loss of EXOSC10 activity, underlining the specificity revealed by our approach. Fourth, the level of other exosome components and the integrity of the exosome are not observably different between *DIS3-AID* cells and parental cells.

While PROMPTs are stabilized by RNAi of EXOSC10 from *DIS3-AID* cells, no effect is observed when EXOSC10-AID is rapidly depleted, even though *bona fide* substrates are stabilized at this early timepoint. Long-term auxin treatment of *EXOSC10-AID* cells does cause a mild increase in PROMPT levels, suggesting that RNAi effects are due to prolonged EXOSC10 depletion. This observation suggests that RNAs, such as PROMPTs, are only occasionally targeted by EXOSC10 or that their slight upregulation is an indirect effect of its long-term depletion. A lack of effect of EXOSC10 on PROMPT (and eRNA) turnover is also underscored by our iCLIP dataset, which showed that their recovery is not enhanced by inactivating EXOSC10 (Figure S5C). Moreover, PROMPTs are not stabilized, even when EXOSC10 is catalytically inactive (Figure S5D). These experiments demonstrate an evolving impact of EXOSC10 loss on transcript levels over time that may have an indirect explanation that should be considered when interpreting data from its long-term depletion.

Our experiments do show some role for EXOSC10 in PROMPT turnover when DIS3 is lost as mislocalization of EXOSC10 occurs when DIS3-AID is depleted and co-depletion of both proteins synergistically enhances PROMPT levels. Given the nucleolar enrichment of EXOSC10, it may be lacking in a large fraction of nucleoplasmic exosome complexes, explaining its limited impact on PROMPTs and other DIS3 substrates. Reciprocally, DIS3 shows relative exclusion from nucleoli, raising the possibility of compartment-specific catalytic complexes (Tomecki et al., 2010). We show that EXOSC10 is not required for MTR4 to associate with the exosome core, as judged by its continued immunoprecipitation with EXOSC2 in auxin-treated *EXOSC10-AID* cells. This is resonant with recent structural data demonstrating that MTR4 contacts the human exosome via MPP6 and EXOSC2 and explains how a lack of EXOSC10 is compatible with the continued degradation of transcripts by DIS3 (Weick et al., 2018).

As it was initially difficult to identify EXOSC10 substrates from our RNA-seq data, we used iCLIP to detect RNAs directly bound by EXOSC10. This was facilitated by using the inactive protein, which revealed that signatures of EXOSC10 bound more robustly than the wild-type protein. There was an obvious predominance of short (∼30 nt) extended precursors to 5.8S rRNA, which we also saw by northern blotting. The sharp reduction of iCLIP reads beyond this 30-nt footprint strongly suggests that EXOSC10 is involved in a final nuclear trimming step, similar to what has been shown in budding yeast (Allmang et al., 1999a). Structural studies lend support to this hypothesis, having shown that bulky RNA particles can become stalled at the entrance to the central channel of the exosome, necessitating a handover from Rrp44 to Rrp6 (Fromm et al., 2017, Schuller et al., 2018). We suggest that handover is also required for human snoRNA processing because short extended snoRNAs are bound by EXOSC10 and stabilized upon its loss and because previous PAR-CLIP shows DIS3 association with longer snoRNA precursors (Szczepińska et al., 2015). As snoRNAs are often present in the introns of expressed genes, stabilized extensions may often be masked by host gene reads in RNA-seq, with iCLIP providing a more direct assessment of their fate. We would also like to note that the exosome may act redundantly with other snoRNA processing pathways in humans (Berndt et al., 2012).

In studying the termination of exosome-sensitive RNAs emanating from protein-coding gene promoters, we found that PROMPTs and some truncated sense transcripts are insensitive to XRN2 loss. Even so, many PROMPTs harbor PASs and poly(A) tails, and XRN2 is implicated in some antisense transcriptional termination by mNET-seq (Nojima et al., 2015). However, the detection of poly(A) tails does not necessarily mean that polyadenylation occurs on every RNA in a population, and it is possible that truncated sense transcripts are generated in multiple ways. A complex consisting of the cap-binding complex and ARS2 is implicated in the 3′ end processing and termination of short human transcripts, including PROMPTs (Hallais et al., 2013, Iasillo et al., 2017). At least some ARS2-sensitive transcripts are generated by mechanisms that do not involve the canonical polyadenylation complex. The differential XRN2 effect on PROMPT and truncated sense transcript termination also suggests a variety of promoter proximal termination processes.

In summary, our data further highlight the constant and rapid turnover of thousands of transcripts in the human nucleus and identify specific substrates for DIS3, EXOSC10, and XRN2. They also reveal that transcripts with apparently similar characteristics (e.g., PROMPTs, PCPA products) can be subtly distinguished on the basis of their sensitivity to XRN2. We anticipate that the ability to rapidly control exoribonucleases, as we have done here, will be especially useful to interrogate processes that cannot be dissected by long-term depletion (e.g., to test the importance of short-lived RNAs and RNA turnover in stress responses or other changes in cellular environments).

## STAR★Methods

### Key Resources Table

**Table:** 

REAGENT or RESOURCE	SOURCE	IDENTIFIER
**Antibodies**		

AID	MBL	Cat# M214-3; RRID:AB_10897312
EXOSC10 (Figure 1B)	abcam	Cat# Ab50558; RRID:AB_869937
EXOSC10 (other figures)	Santa Cruz	Cat# Sc-374595-X; RRID:AB_10990273
DIS3	Bethyl DIS3	Cat# A303-765A; RRID:AB_11205807
Nucleolin	abcam	Cat# Ab22758; RRID:AB_776878
MTR4	Bethyl	Cat# A300-614A; RRID:AB_2187483
GFP	abcam	Cat# ab290; RRID:AB_303395
EXOSC3	abcam	Cat# ab156683; RRID:AB_2619635
MYC	Santa Cruz	Cat# sc-40; RRID:AB_627268
Mouse Alexa Flour 555	Invitrogen	Cat# A-21424; RRID:AB_141780
Rabbit Alexa Fluor 488	Invitrogen	Cat# A-11034; RRID:AB_2576217
CPSF73	abcam	Cat# ab131245; RRID:AB_11156933
EXOSC2	abcam	Cat# ab181211
Tubulin	abcam	Cat# ab7291; RRID:AB_2241126
Anti-FLAG^®^ M2 Magnetic Beads	Sigma	Cat# M8823-1ML; RRID:AB_2637089

**Chemicals, Peptides, and Recombinant Proteins**		

Auxin	Sigma	Cat# I3750-5G-A
Benzonase	Sigma	Cat# E1014-5KU

**Critical Commercial Assays**		

Plasmid mini-prep kit	QIAGEN	Cat# 27106
Gibson assembly mastermix (for cloning)	NEB	Cat# E5510S
GFP-Trap beads	Chromotek	Cat# Gtma-20
QuikChange II XL Site-Directed Mutagenesis Kit	Stratagene	Cat# 200521
QIAquick nucleotide removal kit	QIAGEN	Cat# 28304

**Deposited Data**		

Sequencing data EXOSC10 and DIS3	Gene Expression Omnibus	GSE120574
Sequencing data XRN2	Gene Expression Omnibus	GSE109003
Uncropped blots	Mendeley	http://data.mendeley.com/datasets/jyh2wdyb7z/6
EXOSC10 WT iCLIP Biological replicate 1 & 2	(Macias et al., 2015); Gene Expression Omnibus	GSM1892061 & GSM1892062
EXOSC10 CAT iCLIP data	Gene Expression Omnibus	GSE120574
H3K27ac ChIP-seq	Gene Expression Omnibus	GSE31755
H3K4me1 ChIP-seq	Gene Expression Omnibus	GSE31755
H3K4me3 ChIP-seq	Gene Expression Omnibus	GSE35583
ChIP input control	Gene Expression Omnibus	GSE31755

**Experimental Models: Cell Lines**		

*HCT116:TIR1*	Eaton et al., 2018	N/A
*DIS3-AID*	This paper	N/A
*EXOSC10-AID*	This paper	N/A
*XRN2-AID*	Eaton et al., 2018	N/A
*EXOSC10-AID* + EXOSC10 CAT	This paper	N/A
*EXOSC10-AID* + EXOSC10 WT	This paper	N/A

**Oligonucleotides**		

Control siRNA	Thermofisher	Cat# AM4613
EXOSC10 siRNA	Thermofisher	Silencer select: S10738
qRT-PCR primers	This paper	Table S1
iCLIP Oligos	König et al., 2010, König et al., 2011	Table S2
Northern blot probes	This paper	Table S2

**Recombinant DNA**		

px330 for CRISPR	Cong et al., 2013	Addgene Cat# 42230
GFP-MTR4	(Lubas et al., 2011) A kind gift from the lab of Torben Jensen	N/A
Plasmids for DIS3 tagging	This paper	Critical sequences in Method Details
Plasmids for EXOSC10 tagging	This paper	Critical sequences in Method Details
EXOSC10WT for iCLIP	This paper and (Macias et al., 2015)	N/A
EXOSC10CAT for iCLIP	This paper	N/A
EXOSC10WT for Figure S4A and S4B	This paper	N/A
EXOSC10CAT for Figure S4A and S4B	This paper	N/A

**Software and Algorithms**		

pyCRAC	Webb et al., 2014	N/A
TopHat2	Kim et al., 2013	N/A
BEDTools	Quinlan and Hall, 2010	N/A
ImageJ (colony counting and image processing)		N/A
MACS2	Zhang et al., 2008	N/A
deeptools	Ramírez et al., 2014	N/A
featureCounts	Liao et al., 2014	N/A
DESeq2	Love et al., 2014	N/A
StringTie	Pertea et al., 2016	N/A
SortMeRNA	Kopylova et al., 2012	N/A
HISAT2	Kim et al., 2015	N/A
SAMTools	Li et al., 2009	http://samtools.sourceforge.net/

**Other**		

eRNA & PROMPT annotations	Chen et al., 2016	N/A

### Contact for Reagent and Resource Sharing

Further information and requests for resources and reagents should be directed to and will be fulfilled by the Lead Contact, Steven West (s.west@exeter.ac.uk).

### Experimental Model and Subject Details

Experiments involved human colon carcinoma derived HCT116 cells (male) and human embryonic kidney derived HEK293T cells (female).

### Method Details

#### Cell culture and cell lines

HCT116 and HEK293T were cultured in Dulbecco modified eagle medium with 10% fetal calf serum. Our CRISPR protocol and plasmids was described previously (Eaton et al., 2018). Sequences of EXOSC10 and DIS3 homology arms are provided in this manuscript. Briefly, HCT116 cells grown on a 30mm dish were transfected with 1ug each of guide RNA plasmid, Neomycin and Hygromycin repair constructs. Transfection was with Jetprime (Polyplus) following the manufacturers’ guidelines. Media was changed after 24 hours and, after 72 hours, cells were re-plated into 100mm dishes in media containing 30ug/ml Hygromycin and 800ug/ml Neomycin. Resistant colonies were picked and screened by PCR 10-14 days later. Correct genomic insertion of tags was assayed by sequencing these PCR products. Auxin was used at a concentration of 500uM for one hour unless stated otherwise. For RNAi, 24-well dishes were transfected with siRNA using Lipofectamine RNAiMax (Life Technologies) following the manufacturers’ guidelines. The transfection was repeated 24 hours later and, 72 hours after the first transfection, RNA was isolated.

#### qRT-PCR and 4sU analysis

In general 1ug of RNA was isolated using Tri-reagent and DNase treated for one hour before reverse transcription (Protoscript II) using random hexamers. cDNA products were diluted to 50ul volumes. 1ul was used for real-time PCR in a QIAGEN Rotorgene instrument using Brilliant III SYBR mix (Agilent technologies). The comparative quantitation option in the software was used to generate graphs. The 4sU qRT-PCR protocol is as described in Eaton et al., 2018.

#### Immunofluoresence

Cells were grown on coverslips, treated for 0, 1, 2, 3, or 4 hours with auxin, washed with PBS, fixed for 10 minutes in 4% PFA, washed with PBS, permeabilised with 0.1% Triton x-100 (*v/v* in PBS) for 10 minutes, then blocked with 10% FBS (*v/v* in PBS) for 1 Hour. Cells were probed overnight with 1:1000 diluted α-EXOSC10 and α-nucleolin at 4°C, washed with 0.01% NP40 (*v/v* in PBS), probed with Alexa Fluor® 488 anti-rabbit and Alexa Fluor® 555 anti-mouse secondary’s (1:2000, Invitrogen) for 1 hour, counter stained with DAPI, washed and mounted. All images were taken using an Olympus-81 oil immersion microscope, exposure times, brightness and contrast settings are identical between images.

#### Nuclear RNA-seq

Nuclei were extracted using hypotonic lysis buffer (10 mM Tris pH5.5, 10 mM NaCl, 2.5 mM MgCl_2_, 0.5% NP40) with a 10% sucrose cushion and RNA was isolated using Tri-reagent. Following DNase treatment, RNA was Phenol Chloroform extracted and ethanol precipitated. After assaying quality control using a Tapestation (Agilent), 1 μg RNA was rRNA-depleted using Ribo-Zero Gold rRNA removal kit (Illumina) then cleaned and purified using RNAClean XP Beads (Beckman Coulter). Libraries were prepared using TruSeq Stranded Total RNA Library Prep Kit (Illumina) and purified using Ampure XP beads (Beckman Coulter). A final Tapestation D100 screen was used to determine cDNA fragment size and concentration before pooling and sequencing using Hiseq2500 (Illumina) at The University of Exeter sequencing service. GEO accession numbers: (*EXOSC10-AID* and *DIS3-AID* cell RNA-seq: GSE120574), (*XRN2-AID* cell RNA-seq: GSE109003).

#### RNA-Seq Read Alignment

Raw single-end 50bp reads were screened for sequencing quality using FastQC; adaptor sequences were removed using Trim Galore! and trimmed reads shorter than 20 bp were discarded. All nuclear RNA-seq analyses were carried out using the Ensembl GRCh38.p10 and GRCh38.90 human gene annotations. Before alignment, trimmed reads were passed through the SortMeRNA pipeline (Kopylova et al., 2012) to remove trace rRNA matching in-built 18S and 28S human databases then mapped to GRCh38 using HISAT2 (Kim et al., 2015) with default parameters supplemented with known splice sites. Unmapped, multimapped and low MAPQ reads (< 20) were discarded from the final alignment using SAMtools (Li et al., 2009).

#### *de novo* Transcript Assembly

*de novo* transcripts were assembled from each library using the StringTie suite (Pertea et al., 2016) with default parameters, guided by current GRCh38 reference annotation. Known annotated genes were dropped and the assembled transcripts from each sample were merged into a single consensus annotation. Reads were then counted per transcript using featureCounts (Liao et al., 2013, Liao et al., 2014) and differentially expressed upregulated *de novo* gene intervals (≥2-fold, padj < 0.05) were called using DESeq2 (Love et al., 2014). *de novo* transcripts were designated as a PROMPT (< 3 kb) or eRNA (> 3 kb) based on their relative distance from the nearest annotated gene.

#### Generation of Synthetic Intron Annotation

A custom intron annotation file was produced from GRCh38 by merging all exon intervals derived from each transcript isoform to generate a synthetic transcript representative of every gene. Each synthetic exon was then subtracted from gene intervals using the BEDtools suite (Quinlan and Hall, 2010) producing intron intervals with inherited gene information. Synthetic introns were counted and numbered according to their strand orientation i.e., sense introns numbered ascending, antisense introns descending, finally merging into a single annotation file.

#### Meta Profiling

##### PROMPT and eRNA Analysis

For metagene analysis, expressed protein-coding and ncRNA genes (> 50 reads per gene) were selected and an extended transcriptional window was then applied to each gene to include a 3 kb region 5′ of the TSS and a 7 kb region 3′ of the TES. Overlapping genes and genes that extended beyond chromosome ends were discarded using the BEDtools suite to prevent double read counting. Profiles of these filtered genes were then generated from RPKM normalized reads using deeptools (Ramírez et al., 2014) with further graphical processing performed in the R environment (http://www.R-project.org). Normalized coverage plots (RPKM) were visualized using the Integrative Genome Viewer (IGV) suite. For eRNA meta profiles, no extended window was applied and plots were generated directly from RPKM normalized reads and the *de novo* eRNA annotation file.

#### Peak Calling from ChIP-seq Analysis

ChIP-Seq data was generated by ENCODE from immunoprecipitation (IP) of acetylated histone 3 lysine 27 (H3K27ac) (GEO: GSE31755), monomethylated histone 3 lysine 4 (H3K4me1) (GEO: GSE31755), trimethylated histone 3 lysine 4 (H3K4me3) (GEO: GSE35583) and an input control sample (GEO: GSE31755) in unmodified HCT116 cells. Raw single-end ChIP-seq reads were processed to remove adaptor sequences and low quality reads then mapped to GRCh38 using spliced alignment disabled HISAT2 parameters. BAM alignment files were converted to BED and duplicate reads were discarded and collapsed into a coverage BEDGRAPH file. Peaks were called using MACS2 (Zhang et al., 2008). A background ChIP-Seq signal calculated from the input control sample was compared against each histone modification after sequencing depth normalization, generating a set of peaks for each mark. Peaks were then passed through a Poisson test to call peaks with a qvalue cut-off < 0.05 producing coverage files of peak enrichment. Enrichment of H3K4me1 and H3K4me3 marks were compared and visualized as a log2 ratio using deeptools.

#### Northern Blot Analysis

Total RNA was separated on a 12% Urea-PAGE gel, transferred on to a Hybond-N+ nylon membrane (GE Healthcare), dried and UV crosslinked (2 × 1200 μjoules/cm2) before blocking in hybridization buffer (6x SSPE [150 mM NaCl, 9 mM NaH_2_PO_4_, 1 mM EDTA (pH to 7.4], 5x Denhardt’s Reagent, 0.2% SDS) at 37°C for 1 hour. DNA probes were 5′ radiolabelled with [γ-^32^P]ATP using T4 PNK (NEB) and cleaned with QIAGEN QIAquick nucleotide removal kit. Probes were then added to the hybridization buffer and incubated at 42°C overnight. Membranes were then rinsed in hybridization buffer 3 times for 1 minute then washed at 42°C for 15 minutes before drying and developing on a Phosphor screen. Images were developed on a GE Typhoon FLA 7000 (GE Healthcare). Developed images were then quantitated and analyzed using the ImageJ suite. Membranes were probed with the 5.8S 3′ ext probe first before stripping and re-probing with the mature 5.8S probe.

#### Colony Formation Assay

Cells were seeded into 100 mm cell culture plates and grown in auxin or ethanol (solvent) for 10 days. Growth media and auxin were replaced every 2-3 days. Colonies were fixed in ice cold methanol for 10 minutes and stained using 0.5% (w/v) crystal violet + 25% (v/v) methanol for 10 minutes. Stained colonies were counted using the ImageJ particle analyzer function. Genuine colonies were defined as existing at a density ranging between 50-8000 pixels with a circularity rating between 0.75-1 (1 = perfect circle).

#### iCLIP - Experimental

3xFLAG-EXOSC10^CAT^ was generated from 3xFLAG-EXOSC10^WT^ using Quick-change site-directed mutagenesis kit (Stratagene) to introduce a single amino acid change from Aspartic acid to Asparagine (D313N) within the conserved DEDD-Y motif rendering EXOSC10 catalytically inactive. HEK293T cells were seeded into 15cm plates and transiently transfected with 3xFLAG-EXOSC10^CAT^ and collected 48hrs later when 90% cell confluency was reached. Cells were crosslinked twice on ice using 120 mJ/cm^2^ UVC irradiation, with ice cold PBS replaced after each cross-linking phase. iCLIP was performed on these cell pellets based on the protocol outlined in (König et al., 2011). FLAG-tagged proteins were purified using M2 FLAG Dynabeads. A RNA linker (5′Phosphate-UGAGAUCGGAAGAGCGGTTCAG-3′Puromycin) was ligated to the 3′ end of RNAs, which was described in König et al. (König et al., 2010). Libraries were sequenced using the Illumina HiSeq system (Bejing Genomics Institute).

#### iCLIP - Computational

Reads were demultiplexed, processed and PCR duplicates were collapsed using Flexbar (Dodt et al., 2012), FASTX-Toolkit (http://hannonlab.cshl.edu/fastx_toolkit/), and custom perl scripts, respectively. Biological replicates were combined to increase coverage. Reads were mapped to either Hg38 or a consensus sequence for 45S rDNA using Tophat with the–max-multihits 1 option called. Genome browser files were normalized to reads per million mapped. Average distribution plots for snoRNAs were generated using pyReadCounters.py and pyBinCollector.py from the pyCRAC software package (Webb et al., 2014). iCLIP data for 3xFLAG-EXOSC10^WT^ was obtained from (Macias et al., 2015; GSM1892061 and GSM1892062) and analyzed in parallel with EXOSC10^CAT^ data (GSE120574). SnoRNA table was generated by identifying any snoRNA that had an EXOSC10 iCLIP read mapped within 50nt DS of the 3′ end of a mature snoRNA. PROMPT and eRNA annotations were derived from (Chen et al., 2016).

#### mNET-seq

The mNET-seq experiment and analyses pipeline are as previously published in (Eaton et al., 2018). The XRN2-AID data are deposited with Gene Expression Omnibus (GSE109003).

#### coIP

Approximately 5 million cells were transfected with 5μg of GFP-MTR4 plasmid and the following day, lysed in IP lysis buffer (150mM NaCl, 2.5mM MgCl_2_, 20mM Tris.HCl pH7.5, 1% Triton X-100) by incubation on ice for 30 mins with 1μl of Benzonase. Lysates were clarified by centrifugation (12000rpm for 10 mins) and then incubated with 20μl GFP-TRAP beads (Chromotek) for 1 hour at 4°C with rotation. Beads were washed four times with IP lysis buffer and complexes eluted in 2x SDS gel loading buffer for analysis by western blotting.

#### EXOSC10 HDR 5′

TTGATCCTCCCGCCTTGGCCTCCCAGAGTACTGGGATTACAGGTGTGAGCCACTGCACCCAGCCAAATGTTTTTGTTAAAAACATAAAATCCTAATAATTAAGCCGACCCTGAGGTCAGGGGACTTGCCCGAGGGCAGGAAAAACAGGTCTGCCTTCTCAAGATGCTGCTCAGCTCAGCCAACTCTGGTGGGCCGCCGAGTTCTCTGGGGCCCCTGAGCAAACCATTCTTCCTCTGTTCTGCATGATTAAGATTTGCACCATTTTGTAAACCATCTGAGAACATCCAACCAGCCCGGAAGAAATAACTGTTGTTTTTGTACTCTCTGCAGAGGCTTCAGGTACAACTGGCCACAGAGA

#### EXOSC10 HDR 3′

TAGTCCTGGAAGACACGTGGCGCCTGTGGACCGGAAGCACCAAATGCTGGTGCTGCTTTTGTACATACATATTTTTAAACCATTAAAATTCTTCCTGAAGAAAGCTGATTCCTGACTTTTATTTTGTTGCCGCCACAGCTCTGGCAGGTTGCCATCCTGTTCAGGCAACCATCTTCAGCTGTCTGTGGGCAGGTGAGTGTGCTCCGGGGGTTATGGTGACTTCTAGAAAAATCCAGAGCCGGCCGGGTGCGGTGGCTCACGCCTGCAATCCCAGCACTTTGGGAGGCTGAGGCAGGCGGATCACGAGGTCAGGAGATCGAGACCATGCTGGCCAACACGGTGAAAGC

##### EXOSC10 gRNA target

AGATAGTCCTGGAAGACACG

#### DIS3 HDR 5′

CTTGAAATCAACTCTGATTCTGTCAATCACAGTGGCTCCCCATTGGGAAGGCTGTTTTGTAGTTAAAAAGAACACTTCCTAAATGACATGCTTCTCACCTGTTGAGACCATGTCTAGCTTTTACATTTTTGAACCACTGCTACTTTGTAAAATACCTTCTGTGTATAAAACCTTTAATTAGCCCCCTTTCCCCTCCCTACCACTACATCCTTTTAAATTTGAAGCTGGCAGTGGGGAAGGGGAGGATGAGGTTGAGATGTATTCTATCCTTTAAATCACCTTATTTCCCCCCATTTGCATTACTTTAGATACCAGGAATAAGCATTCCTACAGACACATCTAACATGGACCTTAATGGACCAAAGAAAAAGAAGATGAAGCTTGGAAAA

#### DIS3 HDR 3′

TAGCTATATTCAACAAAAATCTTCAAAGACTGGTTTCTTTTTTAAAAGAAAAAACTTGAAAGAACACTTCTAAGCCTAAGTGTGTGATACAGTTTGTTACTTTTAAGTACATTTTAATAATTTCAGACATCTGCATTTTTATTGAACAGTTGACTGTATCTGACCCATCATACTACTATACTTCTGGGTTGAACAGAATTATTTATGCAGAATAATTCAATTGAATATCCATCACTTAAATACAGTGACAGGACAGCAACTTCAGGGATCTGTAAAGATCATTTAAATGGAGT

##### DIS3 gRNA target

ACTGATACTTCAAACATGGA

#### Codon optimized IAA17 (AID)

GGTAGTGGCATGATGGGTAGTGTGGAGCTGAACCTGCGCGAGACCGAGCTGTGCTTGGGACTGCCTGGCGGCGATACGGTTGCACCCGTTACCGGGAACAAGAGGGGCTTCAGCGAGACAGTGGATCTCAAGCTGAATCTGAACAACGAACCTGCAAATAAAGAGGGAAGCACCACTCATGACGTAGTGACATTCGATAGTAAAGAGAAATCTGCTTGCCCGAAGGATCCAGCTAAGCCCCCGGCCAAGGCCCAGGTGGTGGGATGGCCCCCGGTGCGCTCCTACCGCAAAAACGTGATGGTATCATGCCAGAAAAGCAGCGGGGGGCCCGAAGCCGCCGCTTTTGTTAAAGTGTCAATGGACGGGGCTCCATACCTGAGGAAGATCGATCTCCGGATGTACAAGTCTTACGATGAACTGAGCAACGCGCTTTCAAACATGTTCTCATCTTTCACCATGGGAAAGCATGGGGGCGAAGAAGGAATGATTGACTTCATGAATGAGAGAAAACTGATGGATCTCGTCAATTCTTGGGACTACGTGCCTTCATACGAGGATAAGGATGGAGATTGGATGCTGGTAGGAGACGTGCCTTGGCCCATGTTCGTGGACACTTGCAAAAGGCTCAGACTGATGAAGGGTAGCGATGCCATCGGCTTGGCACCCCGCGCGATGGAGAAGTGTAAATCTAGGGCC

### Quantification and Statistical Analysis

qRT-PCR was quantitated using the comparative quantitation function associated with the QIAGEN Rotorgene instrument. Values were first normalized to ACTB or GAPDH and then samples were compared by quantitating the experimental values relative to the control condition (given the value of 1 by the software). Bars show the average of at least three replicates and error bars show the standard deviation. Where assessed, p values were calculated using a Student’s t test.

### Data and Software Availability

The accession number for the RNA-seq (EXOSC10-AID and DIS3-AID cells) and iCLIP (EXOSC10^CAT^) data reported in this paper is Gene Expression Omnibus: GSE120574.

## Supplemental Information

Document S1. Figures S1–S6 and Tables S1 and S2

Document S2. Article plus Supplemental Information
