# Supplementary material for: Rapid Depletion of DIS3, EXOSC10, or XRN2 Reveals the Immediate Impact of Exoribonucleolysis on Nuclear RNA Metabolism and Transcriptional Control
Source: Cell Rep. 2019 Mar 5;26(10):2779–2791.e5. doi: 10.1016/j.celrep.2019.02.012 (PMC6403362; doi:10.1016/j.celrep.2019.02.012)
Supplement: Document S1. Figures S1–S6 and Tables S1 and S2 [file mmc1.pdf]

Cell Reports, Volume *26*

Supplemental Information

Rapid Depletion of DIS3, EXOSC10, or XRN2 Reveals the Immediate Impact of Exoribonucleolysis on Nuclear RNA Metabolism and Transcriptional Control

Lee Davidson, Laura Francis, Ross A. Cordiner, Joshua D. Eaton, Chris Estell, Sara Macias, Javier F. Cáceres, and Steven West

Davidson et al_ Figure S1

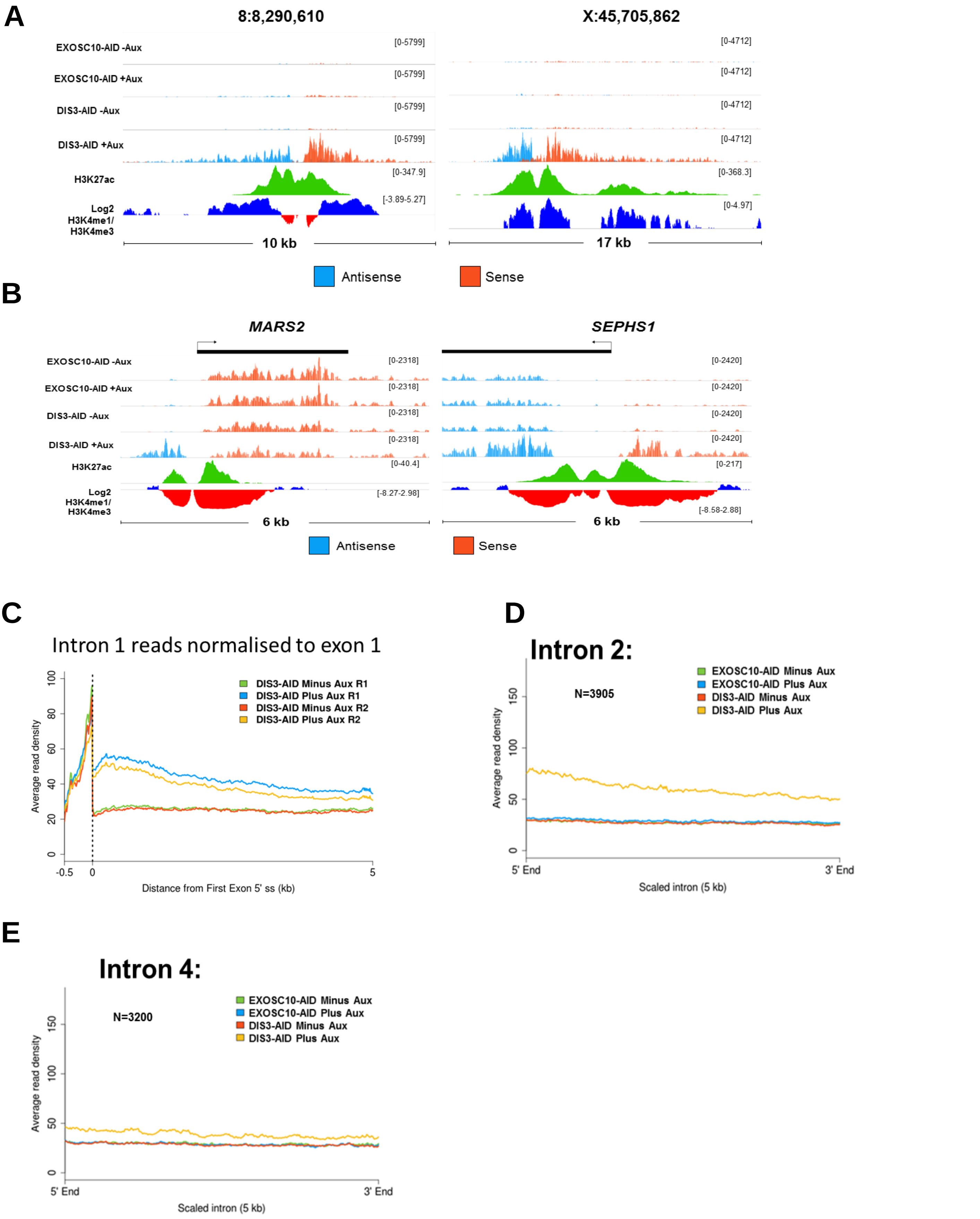

Figure S1: Further analysis of eRNA regions and pre-mRNA stability in *EXOSC10-AID* and *DIS3-AID* cells, related to Figure 3

(A) Examples of eRNA stabilised upon DIS3 loss. These regions show bi-directional transcription (red and blue are signals from opposite strands) and a high level of H3K4me1 versus H3K4me3 characteristic of eRNA transcription. Y-axis shows RPKM for RNA-seq. Lower panel of each figure shows H3K4me1 (blue) vs H3K4me3 (red). H3K4me1 density shown above the line with H3K4me3 below.

(B) Two protein-coding genes shown for comparison with part A. In this case, promoter regions are associated with low H3K4me1 but higher levels of H3K4me3. Scales are as for A. Both eRNA and protein-coding promoters show H3K27ac modification.

(C) Metagene showing enhanced reads over the first intron of genes upon auxin treatment of *DIS3-AID* cell lines. Unlike main text Figure 3F, this representation shows the intron reads normalised to read density in the upstream first exon which was also higher in auxin treated samples possibly because of the general stabilisation of truncated RNAs. For auxin treated DIS3-AID samples, read-density was therefore normalised to the average difference in exonic read-density compared to untreated samples. Finally, both exon and intron metagene profiles were merged into a single profile demarcated by a dotted line. R1 and R2 corresponds to different biological replicates of each experiment.

(D) Metagene showing RNA-seq reads across the second intron of genes in *EXOSC10-AID* and *DIS3-AID* cells treated or not with auxin. The effect of DIS3 loss is diminished relative to the first introns. The plot is not normalised to exon 1 and generated as for Figure 3F.

(E) As for A, but for intron 4. Note, that the effect of DIS3 loss is near absent by this stage in transcription. The plot is not normalised to exon 1 and generated as for Figure 3F.

Davidson et al_ Figure S2

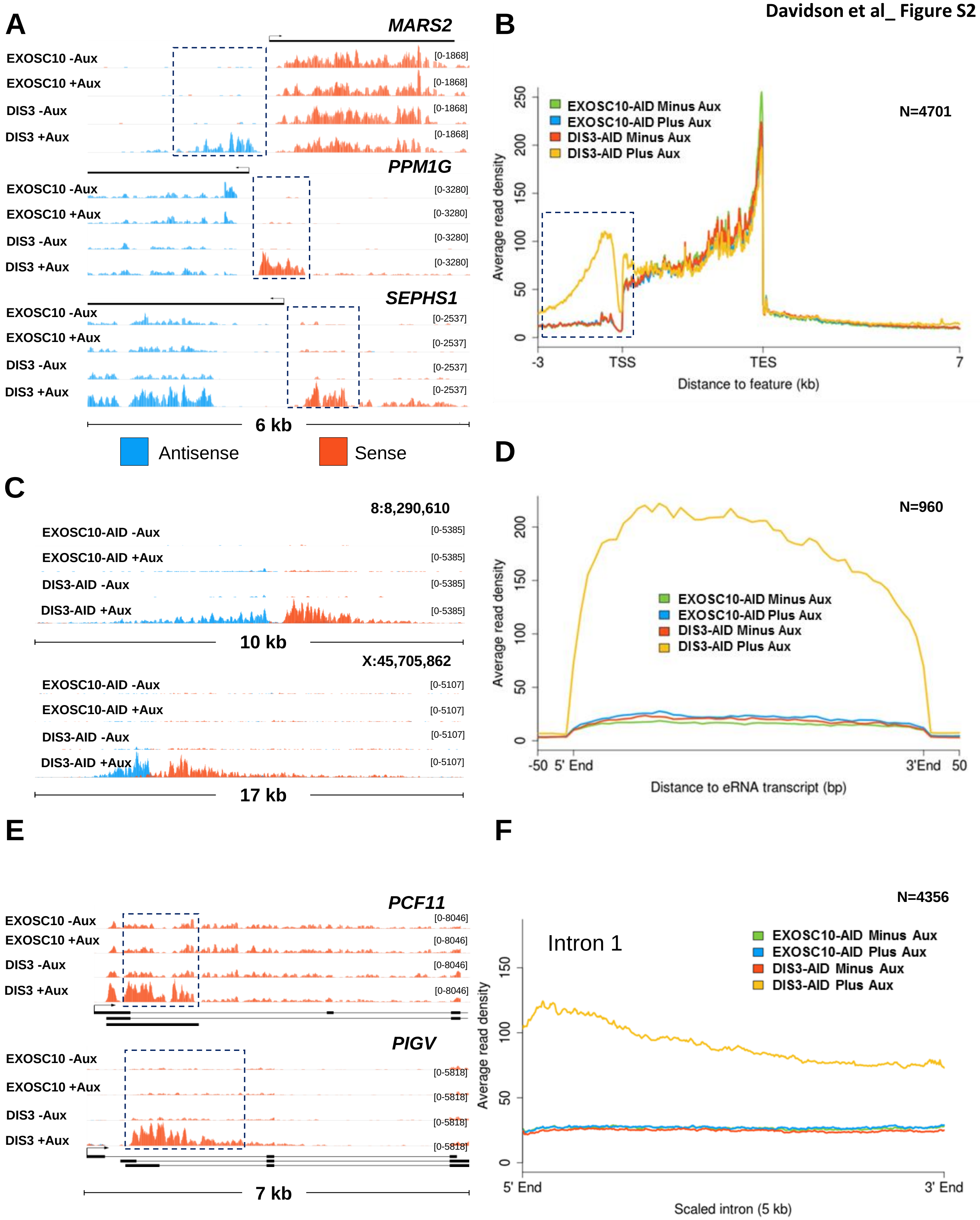

Figure S2: Biological replicate of *DIS3-AID* and *EXOSC10-AID* RNA-seq, related to Figure 3
This figure is the same as main text figure 3 except the data are derived from a second biological repeat of the RNA-seq. Accordingly, annotations are the same as for main-text figure 3.

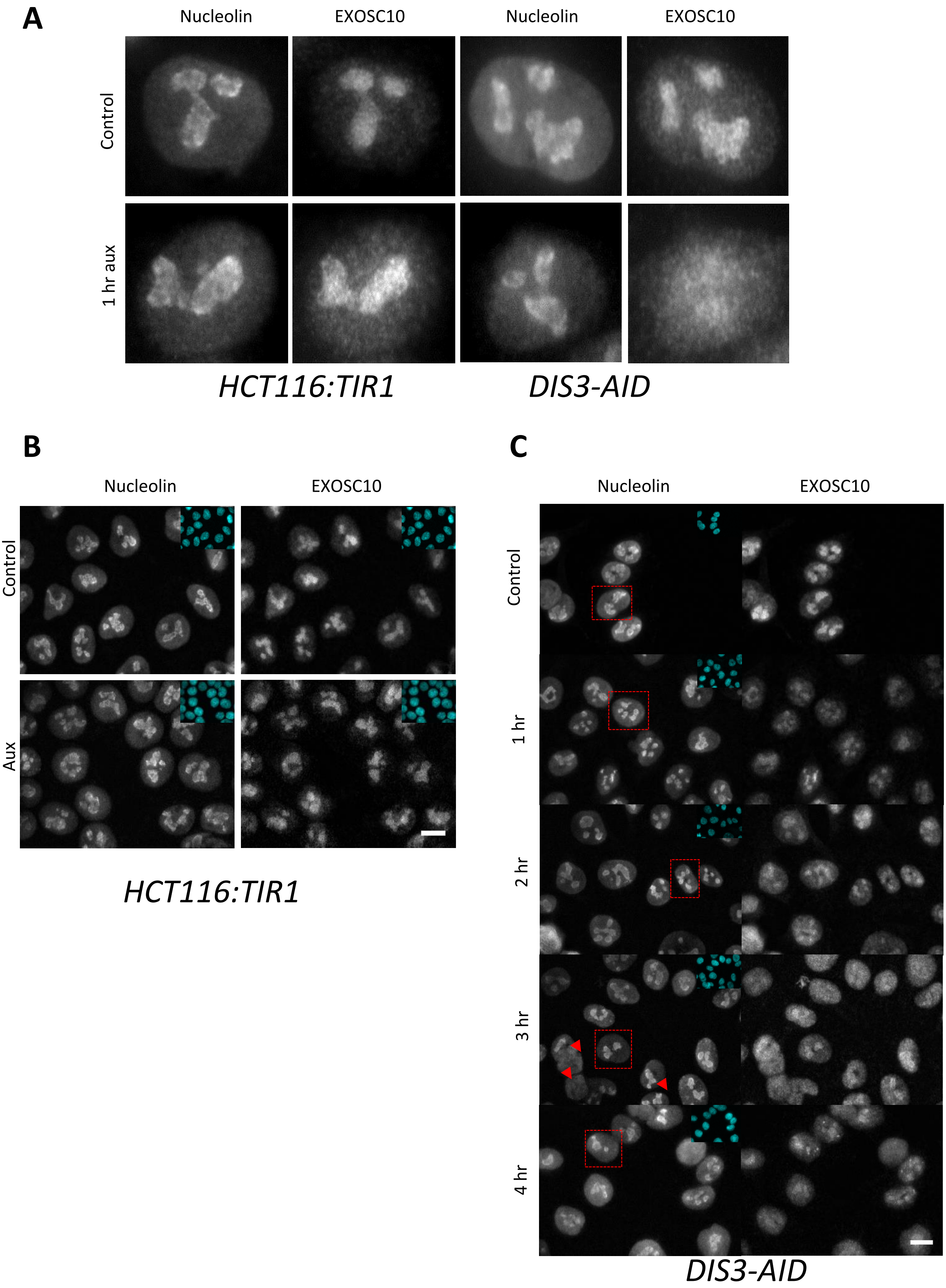
A
Nucleolin
EXOSC10
Nucleolin
EXOSC10
Control
1 hr aux
HCT116:TIR1
DIS3-AID
B
Nucleolin
EXOSC10
Control
Aux
HCT116:TIR1
C
Nucleolin
EXOSC10
Control
1 hr
2 hr
3 hr
4 hr
DIS3-AID

Figure S3: Analysis of EXOSC10 localisation in *HCT116:TIR1* and *DIS3-AID* cells treated or not with auxin, related to Figure 4F

(A) Immunofluorescence experiment whereby *HCT116:TIR1* or *DIS3-AID* cells grown in the presence or absence of auxin (1 hr) are stained for EXOSC10 or nucleolin. This representation is an enlarged view (of a different cell for DIS3-AID to highlight generality) compared to Figure 4F used to further illustrate that DIS3 loss affects EXOSC10 staining in the nucleoli of *DIS3-AID* cells. In contrast, auxin treatment of parental (*HCT116:TIR1*) cells does not impact on EXOSC10 nucleolar staining.

(B) Immunofluorescence experiment whereby *HCT116:TIR1* cells grown in the presence or absence of auxin are stained for EXOSC10 or nucleolin. This experiment confirms the nucleolar location of EXOSC10 and that auxin treatment does not affect this.

(C) A wider field of *DIS3-AID* cells representing the conditions shown in Figure 4F. In each field, the individual cell used in Figure 4F is boxed. Note that 24.6% of cells contain EXOSC10 puncta at the 4h time point (based on a random count of 191 cells, within 13 fields of view across replicates).

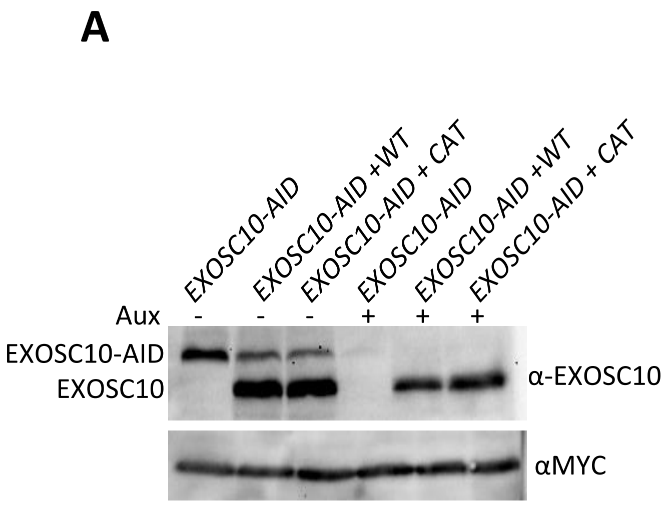
A
EXOSC10-AID
EXOSC10-AID +WT
EXOSC10-AID + CAT
EXOSC10-AID
EXOSC10-AID +WT
EXOSC10-AID + CAT
Aux - - - + + +
EXOSC10-AID
EXOSC10
α-EXOSC10
αMYC

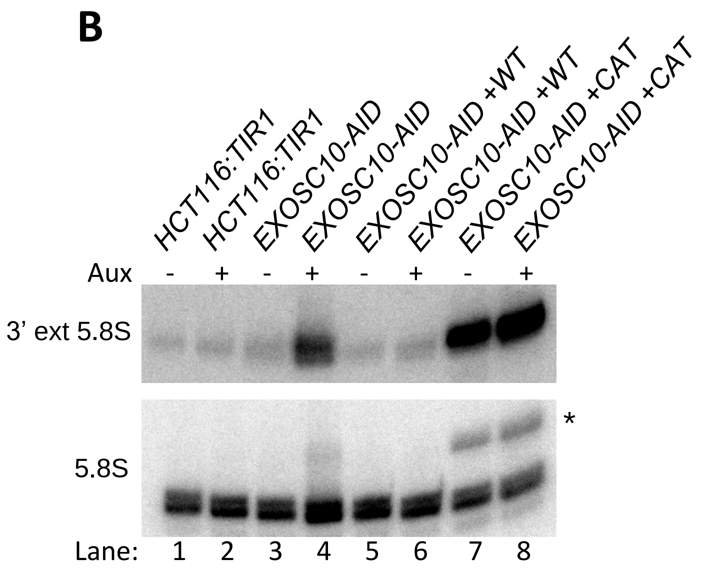
B
HCT116:TIR1
HCT116:TIR1
EXOSC10-AID
EXOSC10-AID
EXOSC10-AID +WT
EXOSC10-AID +WT
EXOSC10-AID +CAT
EXOSC10-AID +CAT
Aux - + - + - + - +
3' ext 5.8S
5.8S
*
Lane: 1 2 3 4 5 6 7 8

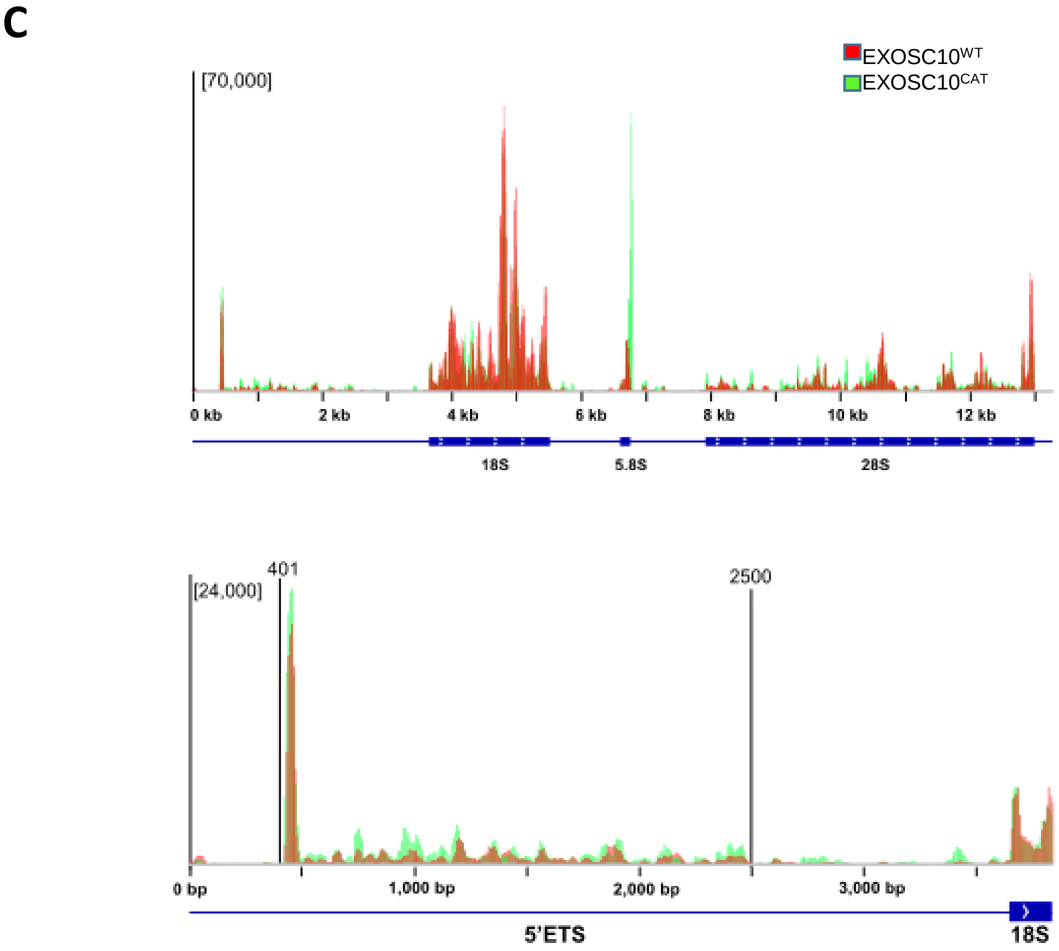
C
EXOSC10WT
EXOSC10CAT
[70,000]
0 kb
2 kb
4 kb
6 kb
8 kb
10 kb
12 kb
18S
5.8S
28S
401
2500
[24,000]
0 bp
1,000 bp
2,000 bp
3,000 bp
5'ETS
18S

Figure S4: Inactive EXOSC10 causes dominant negative stabilisation of rRNA precursors, related to Figure 5

(A) Western blotting of EXOSC10 in *EXOSC10-AID* cells or *EXOSC10-AID* cells stably expressing either wild-type (WT) or inactive EXOSC10 (CAT). In each case, samples show cells treated or not with auxin (1h). This demonstrates the selective depletion of only the AID tagged version.

(B) Northern blotting of the same cell lines and conditions in (A) whereby mature 5.8S rRNA (lower panel) or the 3' extended version (upper panel) were detected. The 3' extended version is also detected by the mature probe (*). Note that expression of inactive EXOSC10 has a dominant negative effect on the accumulation of the 3' extended form, consistent with our iCLIP whereas expressing the WT protein has no such effect (compare lanes 1, 5 and 7).

(C) iCLIP track of 45S rRNA locus showing iCLIP read density obtained from $EXOSC10^{WT}$ or $EXOSC10^{CAT}$. iCLIP reads are obtained across the whole locus but there is a striking accumulation of reads 3' of the 5.8S gene in the $EXOSC10^{CAT}$ sample. Units are reads per million mapped. Lower track is zoomed to the 5'ETS sequence making it clear that there are sites of EXOSC10 binding, some of which are modestly enhanced in $EXOSC10^{CAT}$ samples. Units are reads per million mapped.

A

	SNORD	SNORA	SCARNA	Total
WT	87 (68%)	61(56%)	10(66%)	158(63%)
CAT	122(96%)	95(88%)	10(66%)	227(91%)
Bound snoRNA	127	108	15	250

B

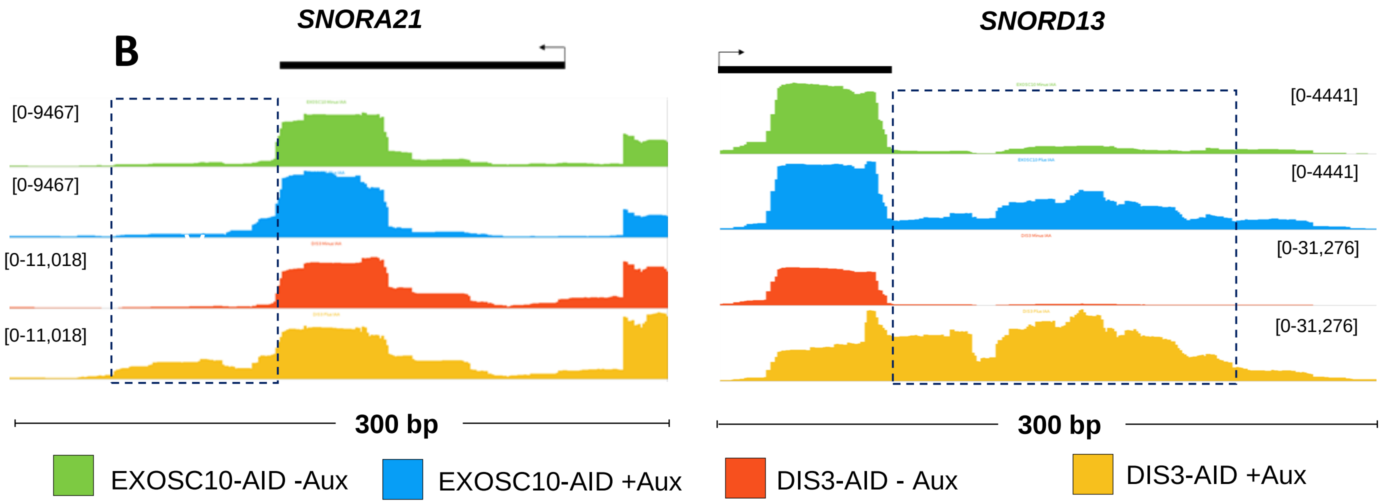

C

	WT %	CAT %	CAT/WT
Total	100	100	1
PROMPT	0.11210626	0.1022381	0.91197516
eRNAs	0.22840467	0.2552155	1.11738286
5' flank snoRNA	0.06893386	0.08152661	1.18267877
Mature snoRNA	3.77523574	2.79753413	0.74102237
3' flank snoRNA	0.14452782	0.30087676	2.08179129

D

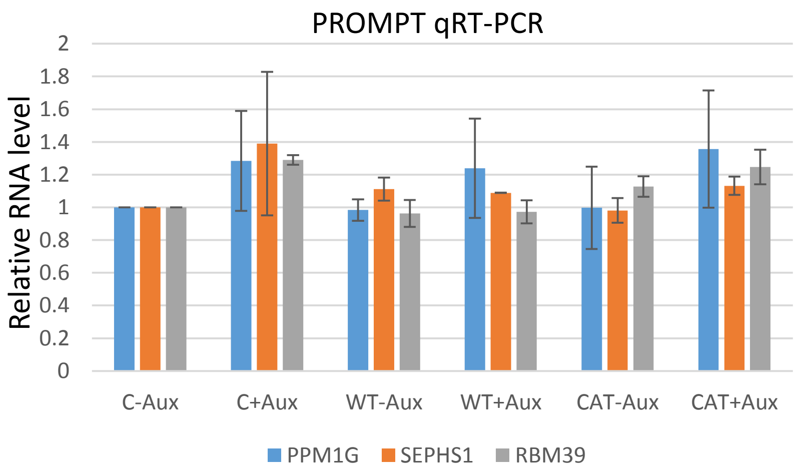

Figure S5: Analysis of snoRNA 3' extensions upon EXOSC10 or DIS3 loss, related to Figure 5

(A) Table summarising the percentage of expressed snoRNAs bound by either $EXOSC10^{WT}$ or $EXOSC10^{CAT}$.

(B) IGV track of SNORA21 and SNORD13 in *EXOSC10-AID* and *DIS3-AID* cells treated or not with auxin. SNORA21 shows a longer 3' extension when DIS3 is depleted and a shorter one when EXOSC10 is lost. SNORD13 also shows extended stabilisation upon loss of DIS3 and, more mildly, EXOSC10. SNORD13 was the only example we found showing longer extensions in both cell lines.

(C) Table showing the proportion of iCLIP reads in the WT and CAT experiment that correspond to PROMPT, eRNA, 5' flank of snoRNA, mature snoRNA or 3' flank of snoRNA. Also shown is the uplift (or not) in the proportion of reads as a result of using the inactive EXOSC10 (CAT/WT). *Bone fide* substrates, exemplified by 3' extended snoRNAs (red box), are more enriched in the CAT experiment. However, PROMPTs and eRNAs are not enriched in this manner arguing that they are not normally EXOSC10 substrates – an observation supported by our RNA analyses.

(D) qRT-PCR analysis of PPM1G, SEPHS1 and RBM39 PROMPTs in *EXOSC10-AID*, *EXOSC10-AID+WT* or *EXOSC10-AID+D313A* cells treated or not with auxin (1h). Values are shown relative to those obtained in untreated *EXOSC10-AID* cells after normalising to GAPDH levels. Error bars are standard deviation.

Davidson et al_ Figure S6

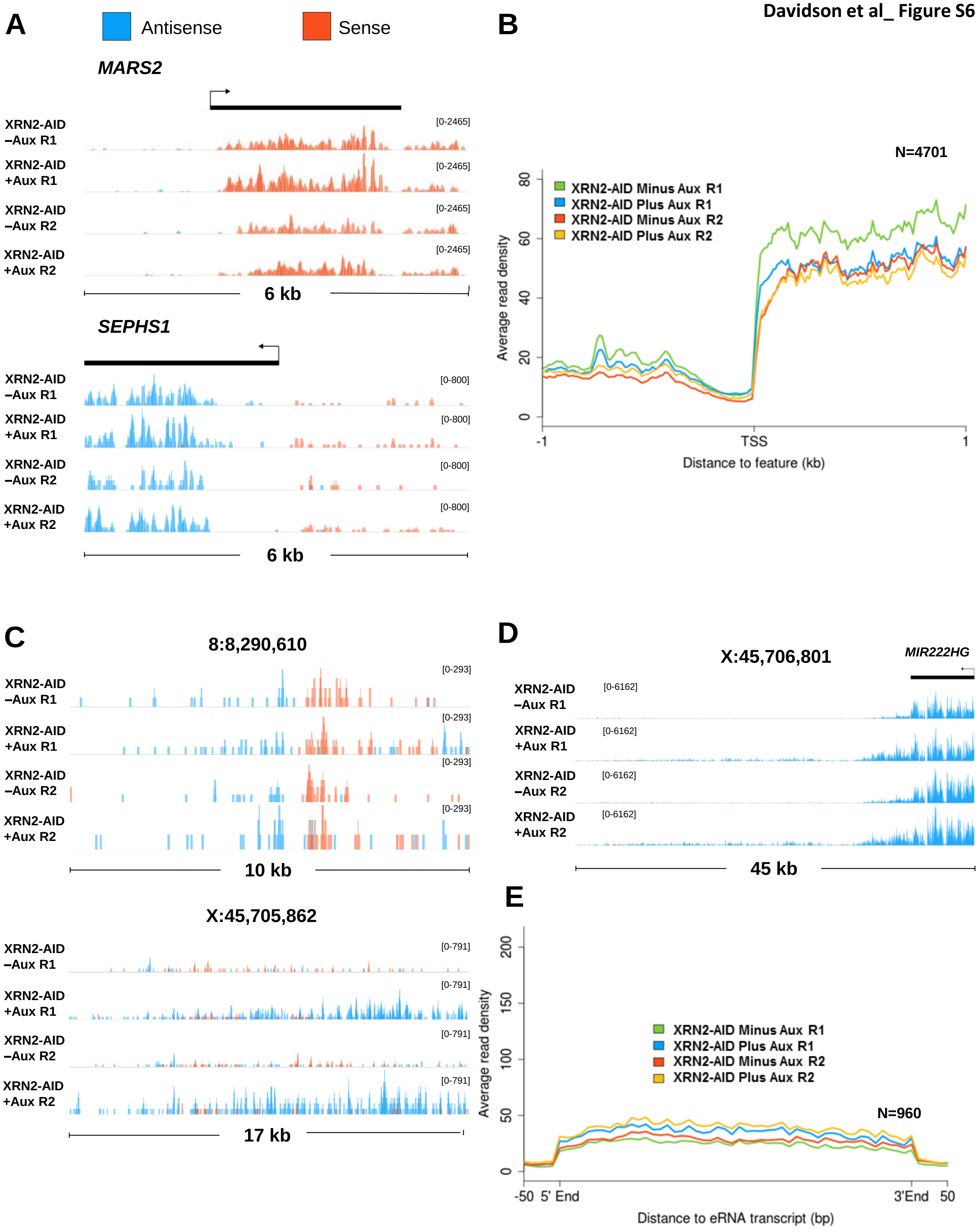

Figure S6: XRN2 plays no role in degrading PROMPT, eRNA or attenuated pre-mRNA, related to Figure 6

(A) *MARS2* and *SEPHS1* PROMPT regions in RNA-seq data obtained from *XRN2-AID* cells treated or not with auxin. Note that there is a lack of XRN2 effect on PROMPT region RNAs (boxed) in both cases. “R1” and “R2” denotes two biological replicates. Y-axis units are RPKM.

(B) Metaplot of promoter regions in RNA-seq data obtained from *XRN2-AID* cells treated or not with auxin. Note that there is a general lack of XRN2 effect on PROMPT region RNAs consistent with our mNET-seq analyses in Figure 6.

(C) Tracks showing the same eRNA regions represented in Figure 3C in RNA-seq data obtained from *XRN2-AID* cells treated or not with auxin. 8:8,290,610 shows no XRN2 effect. Whilst there is an enhanced anti-sense XRN2 effect for X:45,705,862 this is due to a termination defect on a nearby micro RNA expressing gene and not due to eRNA expression (part D of this figure). Note, that we have previously demonstrated that genes with 3’ microRNAs are subject to XRN2-mediated termination (Eaton et al., 2018).

(D) IGV track of *MIR222HG* showing read-through transcription into the X:45,705,862 eRNA region (boxed) when XRN2 is lost. This, not any role of XRN2 in eRNA stability, is responsible for the apparent increase in anti-sense signal seen in part C of this figure. It is also consistent with our previous findings (Eaton et al., 2018) that XRN2 terminates transcription following miRNA processing.

(D) Metaplot of eRNAs in RNA-seq data obtained from *XRN2-AID* cells treated or not with auxin. Note that there is a general lack of XRN2 effect on eRNAs.

Table S1: oligonucleotides used for qRT-PCR, related to STAR METHODS

Amplicon	Forward	Reverse
PIGV US	AAGGGGTTGTTGGGAAGTGA	CCACTTGACCATGCTTGCAT
PIGV DS	AGTTCAGGGCTTCACAGTGA	CTCAGGCCTCTAAACCCCAA
PCF11 US	TGACCATTCTAGCCGAGGAG	GGGGTTGGAGAGAAGGAACA
PCF11 DS	CACACTGGCTCTGCACAAAT	CACACGAACATGCAGGAAGT
PIGV SPL	GACCCTAGAGAAGCCCGATC	CTCAACAGCGGCTCTTGATC
PCF11 SPL	TGAAGAAGAGGAGGAATGGCA	GGGTTCCTGCAATTCGTTTT
GAPDH SPL	GCACCACCAACTGCTTAGC	ATGATGTTCTGGAGAGCCCC
ACTB SPL	CATCCGCAAAGACCTGTACG	CCTGCTTGCTGATCCACATC
PPM1G	GCCTGGCCAAGTACTTCCTA	CCTCTTCCTTCGTTCCCAGT
STK11IP	GGGAGTCTAAGGAAAAGGAG	CAGTGAAAGGAGAGCGTATC
SERPINB8	ACCAAGCAAAGGAGTCAGGA	ACGTCCACCCAAACATTCTC
RBM39	GGAAATAGTGGAGAAAAGCA	CATTTTTGAAGGAACGGTAG
FOXP4-AS	TGCACAATTTCACACCTAGA	ATGTTAGTGACACCTGCACA
SEPHS1 PROMPT	TCTCCACATTTCACGAGGCT	ATTTGCACAGGAGGCTAGGA
DIS3 SPL	ACCCTCACTTAAAATAGAAGATACAGT	CCATTAAGGTCCATGTTTGAAGT
CLIP4 SPL	ATTTTCTGCTTCTGATACCCCA	CCCACTGTCTCAAAATGGCA
CLIP4 US	GTCAGGCTGTTCACGTCATC	TTTCAAAGGCGCCCGTTTTA
CLIP4 DS	TCCTTTGTTTGGAAGATACCCA	GGCGTAACAGAGAAGTCAAGT
SEPHS1 SPL	GGAAACATGTTCGGCCTCAT	CTATGATTCTGGCTGTGCGG
SEPHS1 US	TGAGCGCCTTCCTGATACAA	TTTAACACAGCCTCACCCCT
SEPHS1 DS	GGTGTCATGTGAACCTGCAG	CTGCCAGCGAATCAAGTGAA

Table S2: oligonucleotides used for iCLIP and Northern blot probe sequences, related to STAR METHODS

Name	Sequence
iCLIP Oligo: L3 3’ RNA adapter	5’Phosphate-UGAGAUCGGAAGAGCGGUUCAG-3’Puromycin
iCLIP Oligo: rtCLIP_5	5’Phosphate-NNCGCCNNNAGATCGGAAGAGCGTCGTGgatcCTGAACCGC
iCLIP Oligo: Cut_oligo	GTTCAGGATCCACGACGCTCTTCaaaa
iCLIP Oligo: P5Solexa	AATGATACGGCGACCACCGAGATCTACACTCTTTCCCTACACGACGCTCTTCCGATCT
iCLIP Oligo: P3Solexa	CAAGCAGAAGACGGCATACGAGATCGGTCTCGGCATTCCTGCTGAACCGCTCTTCCGATC
5.8S Northern probe	GCAAGTGCGTTCGAAGTGT
5.8S 3’ EXT Northern probe	GGGGCGATTGATCGGCAA
